# Cryo-EM structures of the XPF-ERCC1 endonuclease reveal how DNA-junction engagement disrupts an auto-inhibited conformation

**DOI:** 10.1038/s41467-020-14856-2

**Published:** 2020-02-28

**Authors:** Morgan Jones, Fabienne Beuron, Aaron Borg, Andrea Nans, Christopher P. Earl, David C. Briggs, Ambrosius P. Snijders, Maureen Bowles, Edward P. Morris, Mark Linch, Neil Q. McDonald

**Affiliations:** 1 Signalling and Structural Biology Laboratory, Francis Crick Institute, NW1 1AT London, UK; 2 Structural Electron Microscopy, The Institute of Cancer Research, SW7 3RP London, UK; 3 Mass Spectrometry Science Technology Platform, Francis Crick Institute, NW1 1AT London, UK; 4 Structural Biology of Cells and Viruses, Francis Crick Institute, NW1 1AT London, UK; 5 Department of Oncology, University College London Cancer Institute, WC1E 6AG London, England UK; 6 Institute of Structural and Molecular Biology, Department of Biological Sciences, Birkbeck College, Malet Street, London, WC1E 7HX UK

**Keywords:** Biochemistry, Cryoelectron microscopy, Diseases

## Abstract

The structure-specific endonuclease XPF-ERCC1 participates in multiple DNA damage repair pathways including nucleotide excision repair (NER) and inter-strand crosslink repair (ICLR). How XPF-ERCC1 is catalytically activated by DNA junction substrates is not currently understood. Here we report cryo-electron microscopy structures of both DNA-free and DNA-bound human XPF-ERCC1. DNA-free XPF-ERCC1 adopts an auto-inhibited conformation in which the XPF helical domain masks the ERCC1 (HhH)_2_ domain and restricts access to the XPF catalytic site. DNA junction engagement releases the ERCC1 (HhH)_2_ domain to couple with the XPF-ERCC1 nuclease/nuclease-like domains. Structure-function data indicate xeroderma pigmentosum patient mutations frequently compromise the structural integrity of XPF-ERCC1. Fanconi anaemia patient mutations in XPF often display substantial in-vitro activity but are resistant to activation by ICLR recruitment factor SLX4. Our data provide insights into XPF-ERCC1 architecture and catalytic activation.

## Abstract (web summary)

The endonuclease XPF-ERCC1 is a key component of the repair machinery to process both intra-strand and inter-strand DNA crosslinks. Here the authors present the cryo-EM structures of DNA-free and DNA-bound human XPF-ERCC1 and find that DNA-free XPF-ERCC1 adopts an auto-inhibited conformation, which is opened up upon DNA binding and they also characterise the biochemical properties of patient-derived XPF-ERCC1 mutations.

## Introduction

Structure-specific endonucleases (SSEs) are found in all branches of life and play crucial roles in genome repair, replication and recombination^1^. These endonucleases act on similar DNA structures with defined polarity but use different catalytic mechanisms. The structurally related XPF/MUS81 family are an important group of human 3′-nucleases that associate to form two active endonuclease heterodimers (XPF–ERCC1 and MUS81–EME1) and a DNA translocase (FANCM–FAAP24) with a pseudo-nuclease architecture^2^. XPF–ERCC1 recognises double-stranded/single-stranded (ds/ss) DNA junctions which have a 3′-ssDNA overhang, nicking the dsDNA backbone to produce a substrate for subsequent steps in DNA repair pathways. XPF–ERCC1 activity is essential for removing helical DNA distortions arising from ultraviolet-induced damage and bulky adducts as part of the nucleotide excision repair (NER) pathway^3^. In this context XPF–ERCC1 nicks the damaged DNA strand 5′ of the lesion at the ds/ss junction of an NER repair bubble. It is also required for interstrand cross-link repair (ICLR), some double‐stranded break repair processes, base excision repair, Holliday junction resolution, gene-conversion and telomere maintenance^4–10^. Mutations in *XPF* and *ERCC1* genes are associated with genetic disorders exhibiting diverse phenotypes. These pathologies are caused by defects in the genome maintenance pathways that involve XPF–ERCC1, including xeroderma pigmentosum (XP), Cockayne’s syndrome, Fanconi anaemia (FA), XPFE progeria and cerebro-oculo-facio-skeletal syndrome^11–15^. The genotype–phenotype correlations of XPF–ERCC1 driven diseases are still poorly understood.

**Fig. 1:**
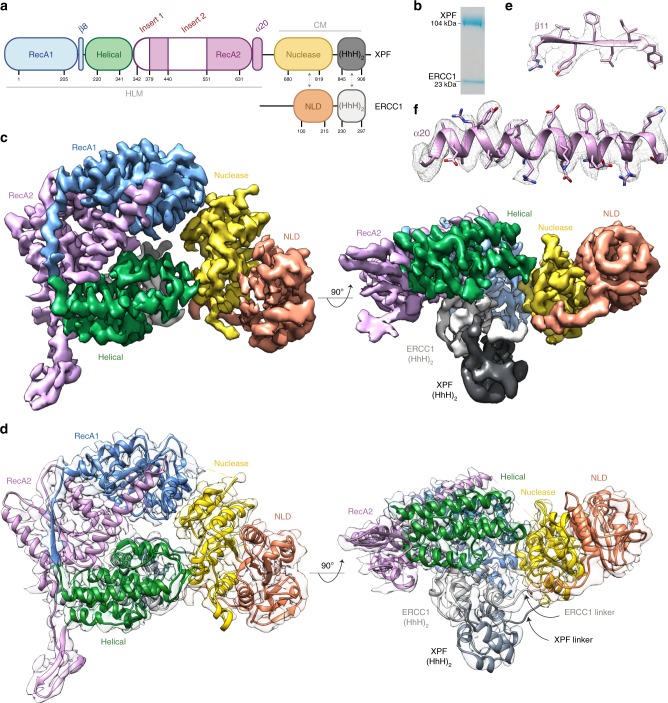
Structure of auto-inhibited human XPF–ERCC1 endonuclease. **a** Domain architecture of XPF–ERCC1 colour coded by domain. XPF: RecA1 (blue), helical (green), RecA2 (pink), nuclease (gold) and (HhH)_2_ (dark grey). ERCC1: NLD (orange) and (HhH)_2_ (light grey). Residue numbering indicates domain boundaries and dotted arrows indicate dimerisation interfaces. Two insert sequences within RecA2 are shown in white embellishing the RecA domain fold. Grey lines define the helicase-like module (HLM) and catalytic module (CM). **b** SDS-PAGE gel of purified recombinant XPF–ERCC1 used for cryo-EM studies. **c** Two orthogonal views of the composite XPF–ERCC1 cryo-EM map ranging from a global resolution of 3.6–4 Å, coloured by domain according to panel a. **d** Final XPF–ERCC1 atomic model coloured by domain according to panel a, displayed within a transparent cryo-EM potential map. The XPF nuclease–(HhH)_2_ and ERCC1 NLD–(HhH)_2_ domain linker is visible at lower map thresholds. **e** Representative region of the cryo-EM map close to strand  ß-11 and sidechains from the final model in pink. **f** Representative part of the cryo-EM map close to α-helix 20 with sidechains shown from the final model in pink.

XPF is the enzymatically active subunit of the heterodimeric XPF–ERCC1 endonuclease and is comprised of a helicase-like module (HLM) and a catalytic module (CM) (Fig. 1a). The XPF HLM is related to the superfamily 2 helicases, with two divergent RecA-like domains that flank an all α-helical domain^16^ (Fig. 1a). Both XPF RecA-like domains, termed RecA-like domain 1 (RecA1) and RecA-like domain 2 (RecA2) lack the residues necessary to bind and hydrolyse ATP^17,18^. Despite this, the HLM is required for full XPF activity and binds both the ICLR recruitment factor SLX4 and ds/ssDNA structures^19,20^. The XPF CM consists of a nuclease domain containing a metal-dependent GDX_*n*_ERKX_3_D active site motif and a tandem helix–hairpin–helix, termed an (HhH)_2_ domain^21^. The smaller ERCC1 subunit has no catalytic activity but is structurally related to the XPF CM, consisting of a nuclease-like domain (NLD) and a dsDNA-binding (HhH)_2_ domain. Both ERCC1 domains heterodimerise with their equivalent domains in the XPF CM, forming discrete nuclease–NLD and 2×(HhH)_2_ functional units. As well as contributing to XPF stability, ERCC1 can recognise ds/ssDNA substrates and engages the XPA repair protein that is required for XPF–ERCC1 recruitment to sites of NER^22^. Currently, there are no available structures of the XPF HLM or of any full-length XPF–Mus81 family members. By solving the structure of a near full-length human XPF–ERCC1 we have defined its overall architecture and uncovered a previously unreported autoregulatory mechanism. We show XPF–ERCC1 adopts an auto-inhibited conformer in the absence of DNA in order to prevent promiscuous cleavage and provide structural evidence for the initial steps of XPF–ERCC1 activation upon binding a DNA junction.

## Results

### Structure determination of human XPF–ERCC1 endonuclease

A single particle cryo-electron microscopy (cryo-EM) density map of purified recombinant XPF–ERCC1 complex (128 kDa) (Fig. 1b) was determined at a global resolution of 4.0 Å (Supplementary Fig. 3a, c, f and Supplementary Movie 1) enabling the assignment of XPF–ERCC1 domain organisation (Fig. 1c, d). The map represents the single dominant conformer observed following 3D classification protocols (Supplementary Fig. 2) and exhibits clear secondary structure features throughout (Fig. 1c and Supplementary Movie 2). Local resolution analysis (Supplementary Fig. 3a) indicated that the heterodimeric 2×(HhH)_2_ domain exhibited some mobility, so signal subtraction of this domain was carried out followed by local refinement. This process improved the global resolution of the resulting sub-volume to 3.6 Å (Supplementary Fig. 3a, d, g) which enabled building, refinement and validation of an atomic model (Fig. 1d). The locally refined map shows clear sidechain density throughout with the local resolution ranging from 3.4 Å in the RecA1 and RecA2 domain cores (Fig. 1e, f) to 7 Å at the periphery of the ERCC1 NLD. Regions modelled as polyalanine or omitted from the final structure are shown in Supplementary Table 1. There is no density recovered for the ERCC1 N-terminus, consistent with it being proteolytically cleaved (Supplementary Fig. 1b). The N-terminus of ERCC1 is not required for wild-type activity in vitro (Supplementary Fig. 1d). Inspection of the angular distribution of assigned particle images during refinement, the 3DFSC curves and 3D flexibility analysis indicate that resolution differences were due to intrinsic flexibility rather than a lack of contributing particle images (Supplementary Fig. 3b, e–g).

### Overall architecture of human XPF–ERCC1 endonuclease

**Fig. 2:**
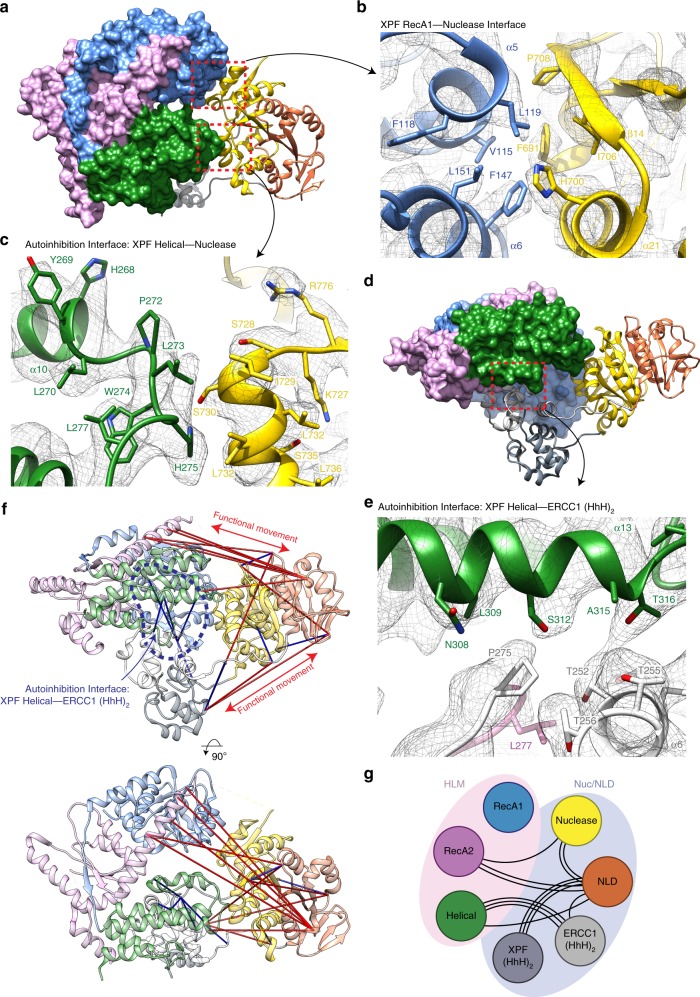
Architecture of the XPF helicase-like module and coupling with the catalytic module. **a** View of the XPF–ERCC1 structure showing the helicase-like module (HLM as surface rendering) contacts the XPF nuclease domain (gold ribbon cartoon) at two interfaces (dashed red boxes). Domains are coloured according to the scheme used in Fig. 1. **b**, **c** Close-up view of interaction interfaces overlaid with the composite cryo-EM map. Selected residues are displayed as sticks and coloured by heteroatom, blue—N, Red—O. **b** The hydrophobic interaction interface between XPF RecA2 (blue) and XPF nuclease domain (gold). **c** Interaction of XPF helical domain residues 273–275 (green) with the XPF nuclease domain (gold). **d** the XPF–ERCC1 HLM (surface rendered) contacts with the ERCC1 (HhH)_2_ domain at a single interface (dashed red box). **e** Interaction of XPF helical domain helix α13 (green) and the ERCC1 (HhH)_2_ domain close to its dsDNA-binding residues (pink). **f** Two orthogonal views of the XPF–ERCC1 structure with XL-MS distance constraints overlaid. Distances within the allowed Cα–Cα cut-off distance of 30 Å are displayed in blue, distances greater than this cut-off displayed in red. Blue dotted line indicates a cluster of allowed distances between the XPF helical and ERCC1 (HhH)_2_ domains. **g** Cartoon schematic representing inter-domain cross-links detected by mass spectrometry. Each black line indicates a single unique cross-link between residues in different domains. Domains within the pink ellipsoid form the XPF HLM, whereas domains within the XPF CM and ERCC1 are within the pale blue ellipsoid.

The cryo-EM structure of near full-length XPF–ERCC1 reveals a compact conformation with extensive interactions between the XPF HLM and CM modules (Fig. 1d). Overall, the HLM adopts a “C”-shape that has dimensions of approximately 70 × 40 × 60 Å. The two RecA-like domains form a rigid platform and lack a nucleotide cleft characteristic of many ATP-driven helicases. Instead the two XPF RecA-like domains are linked through the intimate intertwining of secondary structural elements that extend beyond their globular portion (Supplementary Fig. 4d). While RecA1 caps one edge of the HLM and engages the XPF nuclease domain in the CM, the helical domain caps the other HLM extremity and engages the CM and the dsDNA-binding ERCC1 (HhH)_2_ domain (Figs. 1d and 2a). This arrangement serves to separate and uncouple both functional domains of ERCC1 through its connecting linker. These interactions confirm the key regulatory role for the HLM by engaging crucial elements within the XPF CM and ERCC1. Interfaces observed in the XPF–ERCC1 structure were largely validated using cross-linking mass spectrometry (XL-MS) (Fig. 2f, g) (Supplementary Table 2). Cross-links are found predominately between both the XPF (HhH)_2_ domain and the ERCC1 NLD, and between the XPF RecA2 and ERCC1 NLD. In addition, several cross-links exceeding the distance cut-off are consistent with two principal vectors of dynamic movement in solution.

### Structure of the XPF HLM

The XPF HLM is typical of other helicase superfamily 2 (SF2) members with a RecA1–helical domain–RecA2 organisation, but with substantial inserts within RecA2 (Fig. 1a). In the absence of ATP binding and hydrolysis motifs or a nucleotide binding cleft, RecA1–RecA2 are linked together through a predominantly polar interface (2007 Å^2^). Major interface contributions are made by secondary structural elements ß8 and α20 that form a C-terminal extension to RecA1 and RecA2, respectively, as well as the XPF amino-terminus (Supplementary Fig. 4d). ß8 extends the smaller RecA2 four parallel ß-stranded sheet while α20 packs against the larger RecA1 seven-stranded parallel beta sheet (ß1–ß7). Additional RecA1–RecA2 contacts centre on a π-ring stacking interaction between RecA1 domain Y71^XPF^ and RecA2 domain Y564^XPF^ at one interface edge (Supplementary Fig. 4c) and L39^XPF^ and I592^XPF^ on the other edge. Polar residues make up the remaining contacts with a small cavity. No protein expression was observed for a Y71A^XPF^ mutant (Table 1). The observed structural rigidity of the RecA1–RecA2 unit is structurally homologous to equivalent domains in nucleosome-bound chromatin remodellers ISW1 and INO80^23,24^.

**Table 1:** XPF–ERCC1 mutants that disrupt protein folding.

Protein	Mutant	Mutation rationale	Effect on protein expression
XPF	L608P	XP	Soluble aggregates
XPF	T567A	XP	Soluble aggregates
XPF	R799W	XP	No expression
XPF	Y71A	Structural integrity	No expression
ERCC1	T248A	Autoinhibition	No expression
ERCC1	T252A	Autoinhibition	No expression

XPF RecA2 has two large inserts with unknown functions. Insert one (residues 345–377) separates the helical and RecA2 domains and insert two (residues 441–550) interrupts the RecA2 fold. There is sufficient density in our map to trace the backbone of residues 345–362 and 366–377 from insert one projecting away from the body of the structure. However, no density was recovered for insert two, in agreement with predictions that this region is intrinsically disordered in the absence of DNA. Futhermore, XL-MS data identified a large number of intra-insert cross-links within inserts one and two, consistent with these highly basic regions being flexible (Supplementary Table 2).

The XPF helical domain is an integral part of the HLM and folds as a five anti-parallel helical bundle. This domain packs tightly against RecA2 and is anchored through an interface centred close to residues Q300^XPF^/D302^XPF^ and S412^XPF^/Q419^XPF^ (Supplementary Fig. 4b). The Q300A^XPF^ mutant significantly reduces XPF–ERCC1 expression and increases aggregation (Table 1). Helix α17 (residues 426–440) also contributes to tethering the helical domain to RecA2. The observed position of the helical domain determines the orientation and angle of the extended RecA2 C-terminal α20 helix (Supplementary Fig. 4d), stabilising the HLM conformation through interaction between Q226^XPF^ and T614^XPF^.

### The XPF helical domain regulates XPF–ERCC1 activity

The XPF HLM is coupled to the CM through contacts from RecA1 and the helical domain (Fig. 2a). RecA1 forms a substantial interface (1684 Å^2^) with the XPF nuclease domain involving aromatic and hydrophobic residues from RecA1 α5 and α6 helices and XPF nuclease domain η4 and α21 helices and ß14 strand (Fig. 2b). The hydrophobic nature of the contact suggests that anchoring of the HLM to the XPF nuclease domain through RecA1 forms a permanent part of the XPF–ERCC1 architecture.

The XPF helical domain forms a contact with the XPF nuclease domain that sterically prevents the ds/ssDNA substrate from reaching the XPF active site (Fig. 2c and Supplementary Movie 3). A key contact within this auto-inhibited conformation is between sidechains of H275^XPF^ and S730^XPF^. A H275A^XPF^, W274A^XPF^ double mutant, likely to disrupt this contact, displays a 1.5-fold increase in catalytic efficiency relative to the wild type (Table 2).

**Table 2:** Kinetic data for purified XPF–ERCC1 mutants.

Protein	Mutant	Mutation rationale	*V*_max_ (fmol min^−1^)	*K*_m_ (nM)	*k*_cat_ (s^−1^)	*k*_cat_/*K*_m_ (nM^−1^ min^−1^)
XPF	WT		104.7 ± 2.8	12.1 ± 1.4	20.9 ± 0.66	1.73
XPF	L230R	FA	65 ± 2.1	8.1 ± 1.1	13.0 ± 3.3	1.60
XPF	L236R	FA/CS	91.2 ± 2.5	10.8 ± 1.2	18.2 ± 0.5	1.69
XPF	E239K	FA	88.9 ± 4.7	12.4 ± 2.1	16.9 ± 0.7	1.36
XPF	S786F	ICLR deficient	80.9 ± 3.8	32.5 ± 1.5	7.9 ± 0.8	0.24
XPF	323–326 Δ	ICLR deficient	120.2 ± 1.4	20.5 ± 0.8	24.2 ± 0.3	1.18
XPF	S312A	Autoinhibition	126.1 ± 28.2	5.1	13.5 ± 1.8	2.65
XPF	W274A, H275A	Autoinhibition	193.4 ± 8.6	15.2 ± 2.4	38.7 ± 1.3	2.55
XPF	R112A	DNA binding	24.5 ± 1.1	4.32 ± 0.63	5.6 ± 0.2	1.30
XPF	829–833 Δ	Autoinhibition linker disruption	20.2 ± 0.5	1.9 ± 1.2	3.8 ± 0.2	2.00
XPF	R589W	XP	8.22 ± 2.6	25.9 ± 6.1	1.4 ± 0.9	0.05
ERCC1	L253A	Autoinhibition	33.1 ± 2.7	28.7 ± 22.3	3.4 ± 0.3	0.12

Each kinetic value was obtained from 3 technical replicates (*n* = 3) ± standard deviation (SD).

A second autoinhibitory interface exists between the XPF helical domain and the ERCC1 (HhH)_2_ domain (Fig. 2d, e and Supplementary Movie 3). This interface is formed through predominantly polar contacts involving the highly conserved T248^ERCC1^, T252^ERCC1^ residues and both S312^XPF^ and T316^XPF^. Previous structural and biochemical data suggest that the ERCC1 (HhH)_2_ domain binds dsDNA through hairpin residues S244^ERCC1^–N246^ERCC1^ and G276^ERCC1^–G278^ERCC1^ mainchain atoms^25,26^. These motifs are proximal to T248^ERCC1^ and T252^ERCC1^, and are not accessible in the DNA-free conformation of XPF^25^. The S312A^XPF^ mutant displays a 1.5-fold higher catalytic efficiency than the wild type likely due to the disruption of this autoinhibitory interaction (Table 2). Equally, shortening the connecting linker between the XPF nuclease and (HhH)_2_ domain would be predicted to shift the 2×(HhH)_2_ unit towards the nuclease domain releasing the DNA-binding residues. Indeed, a 829–833Δ^XPF^ mutant displayed a modest 1.2-fold increase in catalytic efficiency and a 7.5-fold tighter *K*_m_ relative to wild type (Table 2).

### Heterodimerisation of XPF and ERCC1 through two interfaces

**Fig. 3:**
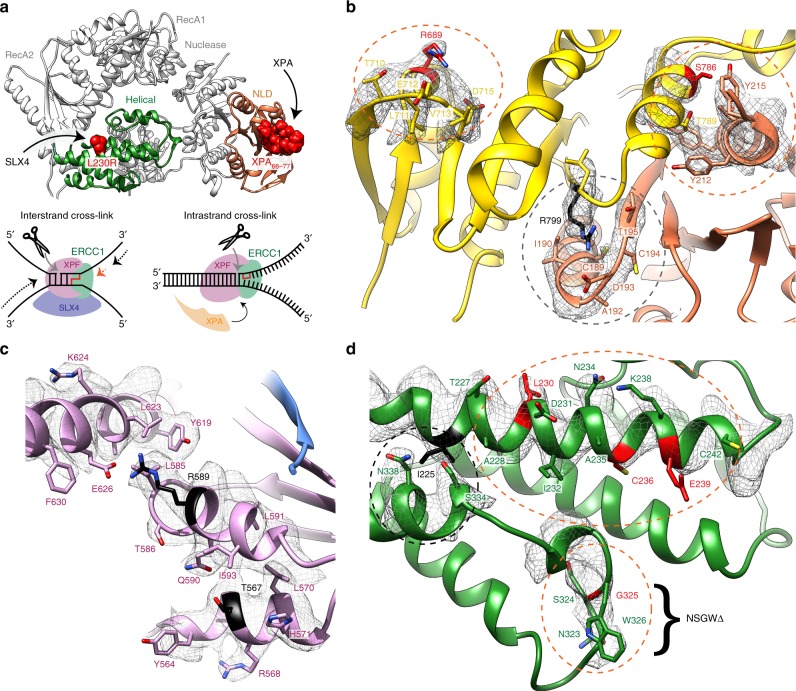
Mapping XPF–ERCC1 disease mutations and DNA repair pathway recruitment sites. **a** Top, a ribbon model of XPF–ERCC1 highlighting the spatially distinct binding sites of XPA and SLX4. XPA binds to the ERCC1 NLD (orange) and SLX4 binds to the XPF helical domain (green). The XPA peptide (residues 66–77) atoms are displayed as red spheres (PDB: 2JNW). The key SLX4 binding residue L230 sidechain atoms are also displayed as red spheres. Bottom, representative DNA structures targeted by XPF–ERCC1 through SLX4 (interstrand cross-link) or XPA (intrastrand cross-link) recruitment. **b**–**d** The molecular environment of patient-derived disease mutations are indicated on the structure, superposed with the cryo-EM map displayed close to the mutation site. Selected residues are displayed using stick rendering coloured by heteroatom. Residues associated with Fanconi anaemia (FA) patient mutations are coloured red whilst those associated with XP are coloured black. Black or orange dashed ellipses indicate the environment close to XP or FA mutations respectively. **b** Mutations in the XPF nuclease domain and ERCC1 NLD lie close to their interface and give rise to both FA and XP. **c** XP-associated mutations disrupt key structural contacts in the XPF RecA2 domain are shown overlaid with the composite cryo-EM map. **d** FA-associated mutations cluster within the XPF helical domain. The helical domain also contains the XP-associated mutant, I225 (black).

ERCC1 is intimately coupled to the XPF CM through two obligate dimerisation surfaces at the equivalent domains of each molecule. The XPF nuclease domain uses a helix–strand–helix motif (α25–ß19–α26) to heterodimerise with the equivalent surface of the ERCC1 NLD (α3–ß8–α4) forming a kidney-shaped dimer with an extensive interaction interface (1684 Å^2^) (Supplementary Fig. 4a). The contact is predominantly hydrophobic and is flanked by three salt bridges (Supplementary Fig. 4a). This interface uses equivalent elements to those mediating heterodimerisation of homologous domains from Mus81–Eme1 and FANCM–FAAP24 complexes^27,28^. We note that the XPF (HhH)_2_ domain hetero-dimerises with the ERCC1 (HhH)_2_ domain through predominantly hydrophobic contacts close to F851^XPF^ and F900^XPF^ as previously observed^26,29^. The (HhH)_2_ domain from XPF and ERCC1 are connected to their XPF nuclease domain/ERCC1 NLD domain through ordered linker sequences. There is sufficient density in our cryo-EM map to trace the mainchain atoms for both linkers (Fig. 1d). The ERCC1 linker makes unexpected interactions with the XPF nuclease domain via Y215^ERCC1^ and D221^ERCC1^ (Fig. 3b). We note that Y215^ERCC1^ lies adjacent to S786^XPF^ suggesting the FA mutation S786F^XPF^ would disrupt this contact with ERCC1. Despite the close association of XPF CM and ERCC1 through heterodimerization, their respective functional domains remain uncoupled and held apart through the extended conformation of their connecting linkers. This is important to consider when comparing with the DNA-bound conformations (see later).

### Structural context of XP and FA patient mutations in XPF

Recruitment of XPF–ERCC1 into either NER or ICLR pathway complexes is dependent on interaction with partner proteins XPA or SLX4 at their respective damaged DNA structures (Fig. 3a). A previous study mapped the XPA-binding site to a cleft within the ERCC1 NLD (Fig. 3a)^30^. This interaction is spatially distinct from the proposed SLX4 site centred within the helical domain at L230^XPF^^19^. Insights from disease mutations have shown that repair pathway recruitment can be disrupted by separation-of-function (FA) or partial loss-of-function (XP) mutations, however the structural basis for this is unclear^31^.

With the availability of a three-dimensional XPF–ERCC1 structure, it was possible to explore the location and structural environment of disease-causing mutations and correlate this with their impact on enzyme stability and catalytic activity. Patient-derived XP or FA-associated mutations were characterised in vitro using a previously reported fluorescence incision assay^20^. Mutations associated with XP mapped primarily to the XPF RecA2 domain and its inserts^15,32,33^. L608^XPF^, R589^XPF^ and T567^XPF^ are located in the folded region of the RecA2 domain, with the latter two forming structurally important intra-domain contacts^32^ (Fig. 3c). Indeed, L608P^XPF^ and T567A^XPF^ mutant proteins formed soluble aggregates when expressed recombinantly, as measured by analytical size exclusion chromatography (SEC) and an R589W^XPF^ mutant exhibited 35-fold reduction in catalytic efficiency (Table 2). The R799W^XPF^ XP mutation failed to express recombinantly and lies on the periphery of the heterodimeric nuclease–NLD interface with ERCC1 (Fig. 3b). These data, taken in the context of our structure, suggest the L608P^XPF^, T567A^XPF^, R589W^XPF^ and R799W^XPF^ XP disease mutants compromise XPF–ERCC1 structural stability (Table 1). I225^XPF^ is also associated with XP^32^ and maps onto the hydrophobic core of the helical domain (Fig. 3d) suggesting it is also likely to contribute to XPF–ERCC1 structural integrity.

FA patients are proficient in NER but deficient in ICLR, indicating a likely separation of function^19,34^. Our structure indicates the FA point mutations within XPF such as L230R^XPF^, C236R^XPF^ and G325E^XPF^ cluster within the XPF helical domain (Fig. 3d)^11^. These mutants, when expressed recombinantly, were found to have a similar level of endonuclease activity to wild-type XPF–ERCC1 against a stem–loop substrate (Table 2). Previous studies indicated these FA mutations are unable to engage SLX4^19^. This would impact both the ability of SLX4 to stimulate XPF–ERCC1 activity^35^ as well as recruit XPF–ERCC1 to ICLR sites in vivo^19^. We found that XPF–ERCC1 co-expressed with a truncated form of human SLX4 (XPF–ERCC1–SLX4^NTD^) indeed showed a six-fold increase in catalytic efficiency (Table 3 and Supplementary Fig. 9a–e). To confirm whether FA XPF–ERCC1 mutant 323–326Δ^XPF^ had a reduced SLX4 association and/or a negative impact on activity, we measured the amount of XPF–ERCC1 endonuclease activity recovered after affinity purification followed by gel filtration. The 323–326Δ^XPF^ FA mutant showed substantially less endonuclease activity (Supplementary Fig. 9d). The FA mutant L230R^XPF^ lies close to XPF residues 323–326 and was previously shown to be unable to bind full-length SLX4, indicating that it forms a key determinant of the SLX4 binding site^19^. Our data are consistent with a differential impact of XPF mutants (loss-of-function) affecting NER from those XPF mutations (separation-of-function) that impact SLX4-driven activation and interaction in ICLR^36^.

**Table 3:** SLX4_1–758_–XPF–ERCC1 mutation data summary.

Protein	Mutant	*V*_max_ (fmol min^−1^)	*K*_m_ (nM)	*k*_cat_ (min^−1^)	*k*_cat_/*K*_m_ (nM^−1^ min^−1^)
XPF(–ERCC1–SLX4^NTD^)	WT	219.2 ± 9.3	4.2 ± 0.7	43.8 ± 1.1	10.4
XPF(–ERCC1–SLX4^NTD^)	323–326 Δ	49 ± 6.5	32.2 ± 9.6	9.8 ± 1.4	0.30
XPF(–ERCC1)	WT	104.7 ± 2.8	12.1 ± 1.4	20.9 ± 0.66	1.73

Each kinetic value was obtained from 3 technical replicates (*n* = 3) ± the standard deviation (SD).

### XPF–ERCC1 conformational activation on DNA-junction binding

We hypothesised that the autoinhibitory interactions formed by the XPF helical domain need to be released following XPF–ERCC1 DNA-junction engagement, prior to the incision reaction. To probe the nature of such potential conformational changes, we assembled a complex of XPF–ERCC1 bound to a DNA stem–loop model substrate (10-duplex 20-T single-strand stem–loop) that we previously showed presents a single incision site to XPF–ERCC1^20^. Using an electrophoretic mobility shift assay (EMSA) we observed 1:1:1 stoichiometric binding of the stem–loop DNA to XPF–ERCC1 (Supplementary Fig. 5b, c).

This sample was used for cryo-EM data collection leading to a single-particle cryo-EM density map at a global resolution of 7.7 Å (Supplementary Fig. 6b). Signal subtraction of the dimeric 2×(HhH)_2_ domain and DNA density, followed by local refinement, improved the resolution of the resulting sub-volume to 5.9 Å (Supplementary Fig. 6c). The locally refined map shows evidence of helical features, with the local resolution highest in the core of the RecA domains (Supplementary Fig. 6a). 3DFSC (Supplementary Fig. 6e, f and Supplementary Movie 4) analysis indicates that the map does not suffer heavily from anisotropy and the lower resolution of the DNA-bound map relative to the DNA-free is as a result of increased flexibility. Indeed, XPF–ERCC1 does not engage DNA in vivo unless recruited by XPA in complex with TFIIH^37^. It is likely that the DNA-bound XPF–ERCC1 complex only becomes fully stabilised in the presence of these additional factors.

**Fig. 4:**
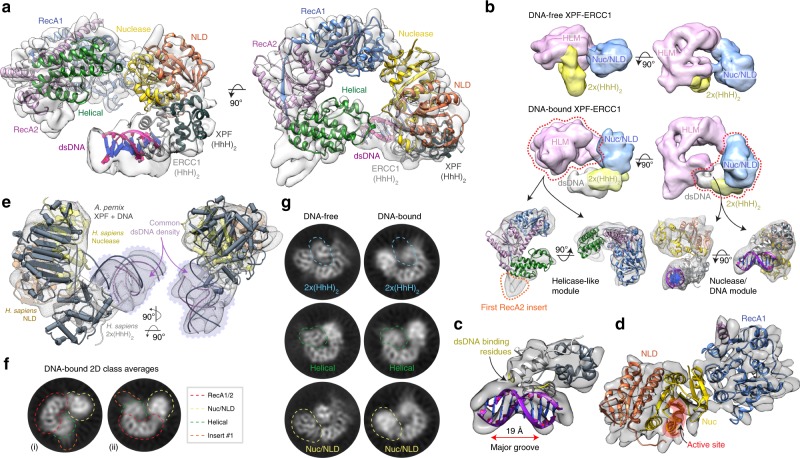
Conformational reorganisation of XPF–ERCC1 engaged by a DNA-junction substrate. **a** Two orthogonal views of DNA-bound XPF–ERCC1 ribbon structure coloured by domain according to Fig. 1a. The dsDNA duplex is shown in purple. The ribbon model is shown with the cryo-EM composite map, ranging from 5.9–7.7 Å global resolution. **b** Top and middle: two orthogonal views comparing segmented DNA-free and DNA-bound maps. The DNA-bound map displayed is the globally refined and unsharpened 7.7 Å map. The DNA-free map displayed is the globally refined and unsharpened 4.0 Å map low-pass filtered to 9 Å resolution to display comparable levels of detail to the DNA-bound map. Both maps were segmented in UCSF Chimera revealing sub-volumes for the XPF HLM (pink), XPF nuclease–ERCC1 NLD dimer (blue), 2×(HhH)_2_ domain (yellow) and dsDNA (white). Bottom: two orthogonal views of the HLM and the CM/ERCC1 dsDNA-binding module. Each sub-volume contains the ribbon model of DNA-bound XPF–ERCC1, orange dotted line indicates unmodeled density corresponding to the position of the first RecA2 domain flexible insert. **c** Fitted model for the dimeric 2×(HhH)_2_ domain engaging dsDNA via the minor groove, placed within map density. Major groove distance and dsDNA-binding residues are indicated. The dsDNA-binding hairpin residues of ERCC1 are highlighted in yellow. **d** Model for the XPF RecA1–nuclease/ERCC1 NLD interface following substrate engagement. Labels and red circles indicate the XPF active site location. **e** Human XPF nuclease–ERCC1 NLD dimer, 2×(HhH)_2_ domain and dsDNA positioned within the cryo-EM map together with a structurally superposed *A. pernix* XPF structure (PDB code 2BGW) bound to dsDNA. A similar dsDNA trajectory is evident (purple box). **f** 2D class averages of DNA-bound XPF–ERCC1 with coloured dotted lines indicating the position of domains according to the key. **g** Comparison of 2D classes of the same molecular views from DNA-free (left column) and DNA-bound (right column) XPF–ERCC1 with coloured circles indicating the position of key domains, coloured according to panel (**f**).

The DNA-bound reconstruction enabled the placement of all XPF–ERCC1 domains using the DNA-free structure as an initial template (Fig. 4a and Supplementary Movie 5). Aligning the DNA-bound and DNA-free maps identified key changes in the architecture of XPF–ERCC1, the most dramatic being the disengagement of the 2×(HhH)_2_ domain from the XPF helical domain and it’s repositioning adjacent to the XPF nuclease—ERCC1 NLD dimer, as seen for other XPF/Mus81 family endonucleases^27,28^ (Fig. 4e). An additional region of density was identified adjacent to the 2×(HhH)_2_ domain but segmented into a distinct volume (Fig. 4b). This density was assigned as the duplex portion of the stem–loop substrate due to the unambiguous presence of a 19 Å concave major groove and its length measuring the distance of 10 base pairs (Fig. 4c). In order to correctly position the 2×(HhH)_2_ domain with respect to the dsDNA, the structure of the *Aeropyrum pernix* XPF homodimer in complex with dsDNA was fit into the map and used to align the human 2×(HhH)_2_—dsDNA functional unit (Fig. 4b, c). The fit to density was then optimised for the human structure using Flex-EM^38^. This positions the 2×(HhH)_2_ domain–dsDNA-binding residues S244^ERCC1^–N246^ERCC1^ and G276^ERCC1^–G278^ERCC1^ in close proximity to the dsDNA minor groove in a homologous fashion to other family members (Fig. 4e). Furthermore, comparison of the DNA-free and DNA-bound 2D class averages clearly indicates a repositioning of the 2×(HhH)_2_ domain upon substrate engagement (Fig. 4a).

The remaining domains of XPF–ERCC1 can be fit unambiguously into the density. The RecA1–RecA2 unit remains structurally rigid, with high-resolution features present in 2D class averages (Fig. 4f, g), reaffirming its role as an inactive helicase. Whilst the remainder of the complex increases in flexibility upon substrate engagement (Fig. 4g), the interface between the XPF RecA1 and nuclease domains remains intact (Fig. 4d). Comparison with the DNA-free structure reveals that the XPF helical domain pivots by approximately 15°, rotating ~11 Å away from the nuclease domain (Supplementary Fig. 7a). The increased flexibility of the XPF helical domain following its disengagement with the XPF nuclease domain can be visualised by the loss of high-resolution features in 2D class averages following substrate engagement (Fig. 4g). This conformational change breaks the autoinhibitory contact formed between H275^XPF^ and S730^XPF^ as predicted from the DNA-free structure. The remaining unmodeled map density likely corresponds to the flexible first RecA2 domain insert (Fig. 4b).

### A model for DNA junction-based activation

**Fig. 5:**
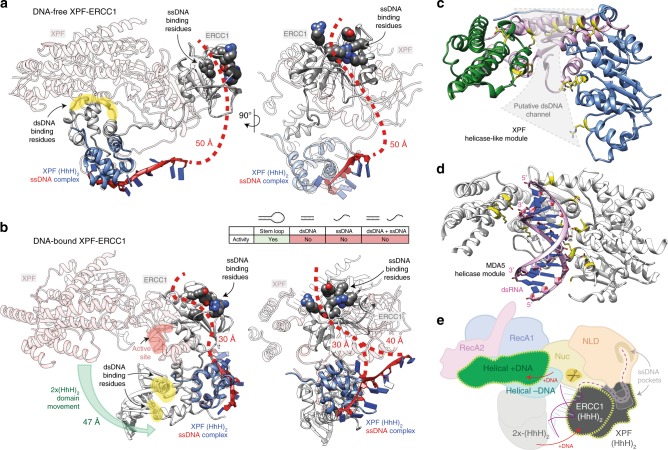
Comparison of DNA-free and DNA-bound XPF–ERCC1 gives insights into DNA-junction activation. **a** Two orthogonal views of DNA-free XPF–ERCC1 highlighting known ssDNA binding sites. An NMR structure of XPF (HhH)_2_ domain bound to a ssDNA (PDB: 2KN7) mapped on to the full-length DNA-free structure indicates a distance of ~50Å between the ssDNA-binding site and residues known to bind ssDNA in the ERCC1 NLD. **b** Equivalent views as in panel a for DNA-bound XPF–ERCC1 indicating the 2×(HhH)_2_ domain undergoes a substantial movement to engage the ERCC1 NLD. This positions the two ssDNA-binding sites close enough to simultaneously engage ssDNA. Inset shows only a stem loop is a substrate for XPF-ERCC1. **c** The XPF HLM from the DNA-bound structure contains a concave surface lines with basic residues (yellow) that could potentially bind to dsDNA. **d** Equivalent view to panel c of the helicase module from MDA5 bound to A-form dsRNA (PDB: 4GL2) through an equivalent positively charged concave surface. **e** Proposed model for XPF–ERCC1 domain rearrangements upon binding stem–loop DNA. Red arrows indicate direction of domain rearrangements from DNA-free to DNA-bound. Dotted yellow lines indicate domains that undergo significant conformational changes. Scissors indicate the approximate location of the active site.

Tight regulation of endonuclease catalytic activity is needed to prevent inappropriate DNA cleavage. Indeed XPF–ERCC1 displays no activity towards DNA duplexes, ssDNA or an equimolar mixture of ds and ssDNA substrate (Fig. 5b). This implies that it is the proximity of the ssDNA and dsDNA elements in a junction context that is uniquely required to stimulate XPF–ERCC1 activation and overcome complex autoinhibition. Analysis of our DNA-bound structure reveals that the presence of a junction shifts the dimeric 2×(HhH)_2_ domain by 47 Å to contact the XPF nuclease–ERCC1 NLD dimer, disrupting contacts with the XPF helical domain (Fig. 5a, b, Supplementary Movies 9 and 10). In this configuration the dimeric 2×(HhH)_2_ domain lies proximal to the ERCC1 NLD domain, coupling both known ssDNA-binding elements of the endonuclease^25,27,28,39^ within the ERCC1 NLD and XPF (HhH)_2_ domain (Fig. 5a, b). Others have proposed that XPF–ERCC1 2×(HhH)_2_ domain is sufficient to recognise ds/ssDNA junctions^40^, however, the precise arrangement of multiple ssDNA and dsDNA domains required for DNA-junction recognition remains to be determined. The final DNA-bound model lacks the single-stranded portion of the stem–loop and places the scissile phosphodiester bond approximately 15 Å from the XPF active site motif (residues 725–727) (Fig. 5b). We interpret the DNA-bound structure as showing important features of an initial step towards full DNA-junction recognition prior to the incision reaction. The low resolution of the DNA component within the cryo-EM map (approximately 9 Å) suggests that the dimeric 2×(HhH)_2_–DNA complex can adopt multiple conformers. Equally, the accessibility of the dsDNA major groove opposite to the 2×(HhH)_2_ minor groove interaction could be re-oriented towards the positively charged concave surface within the XPF HLM (Fig. 5c, d).

The closest structural homologue of both DNA-bound and DNA-free structures, as identified by the DALI protein structural comparison server^41^, is the helicase/translocase MDA5 that binds dsRNA^40–42^ (rmsd of 4.1 Å over 283 C-alphas) (Fig. 5d and Supplementary Fig. 7b, c). MDA5 binds to the major groove of A-form dsRNA using a concave surface lined with basic residues and sequences equivalent to the XPF RecA2 insert two spanning residues 441–550 (Fig. 5d). A similar positively charged concave surface is evident for XPF HLM. Additional density is apparent adjacent to the RecA2 ß-sheet and could represent part of the missing insert two (disordered in the DNA-free structure), and is analogous to a dsRNA-binding region of MDA5. In the absence of DNA, the concave surface of the auto-inhibited conformation of the XPF HLM is too narrow to accommodate dsDNA, however. Upon release of the autoinhibitory contact between the XPF helical and nuclease domains following substrate engagement the HLM opens up into a conformation more conducive to dsDNA major groove binding. Further experiments using substrates with longer dsDNA regions or with A-/B-form DNA duplexes are required in order to validate this proposed mode of binding (Supplementary Fig. 8c).

Superposition of an XPF (HhH)_2_ domain bound to ssDNA (PDB: 2KN7) with our DNA-free structure reveals that the distance between the ssDNA-binding sites on the XPF (HhH)_2_ domain and the ERCC1 NLD is too far (>50 Å) to be engaged simultaneously by the 20-thymine residue stem–loop (Fig. 5a). Movement of the 2×(HhH)_2_ domain in the presence of a stem–loop shortens this distance to approximately 30 Å (Fig. 5b) This is consistent with changes in (HhH)_2_ domain position and linkers observed in published structures for *A. pernix* XPF and Mus81–Eme1 in the presence and absence DNA. It is also supported by both our 3D variability analysis (Supplementary Movies 6–8) and by XLMS data. We therefore speculate that longer junction substrates may reveal even further dynamic rearrangements sufficient to place a junction at the nuclease active site (Fig. 5e).

## Discussion

The structural and functional studies described in this report provide insights into XPF–ERCC1 architecture, regulation and activation. The XPF–ERCC1 endonuclease catalyses the first irreversible step in NER repair by nicking the 5′-edge of the repair bubble structure on the damaged strand. The structure of DNA-free XPF–ERCC1 reveals how the heterodimer is auto-inhibited by blocking both DNA binding and active site access through contacts with the XPF helical domain. This structure reveals inter-domain interfaces not previously described and rationalises our previous report that the HLM impacts on endonuclease activity and substrate interaction^20^. Whilst the ssDNA-binding surfaces of XPF (HhH)_2_ and ERCC1–NLD are fully solvent accessible in the auto-inhibited structure, they are uncoupled from their respective dsDNA-binding surfaces (ERCC1 (HhH)_2_ and XPF–HLM), which are sterically blocked. The structure also confirms the presence of a heterodimeric interface between the XPF nuclease and ERCC1 NLD as described for other family members^25,27,28^.

This study provides evidence linking conformational activation of XPF–ERCC1 through DNA-junction recognition, with a likely contribution from recruitment partner proteins at DNA-junction sites prepared for either NER or ICLR pathways. Mapping the XPA interaction site within ERCC1^30^ and the SLX4 site within XPF helical domain reveals spatial separation of each recruitment partner site in the auto-inhibited state. It suggests the critical binding determinants are non-overlapping, but full structures of XPF–ERCC1 with SLX4 or XPA combined with competition binding studies are required to prove this. XPF–ERCC1 activation by SLX4 is disrupted by some FA mutations that map to the helical domain, in agreement with previous in vivo work^19,34^. Given its proposed regulatory role, the helical domain may be repositioned on binding SLX4 to stimulate activity^35,43^. In contrast, XP-associated mutations were found to generally reduce endonuclease activity in vitro towards an NER substrate by destabilising the complex whereas FA mutants exhibited activity similar to wild type. Interestingly, our XPF–ERCC1 preparations were found to contain a significant amount of active XPF–ERCC1 heterotetramer (Supplementary Fig. 1a, c, d). Cryo-EM data was collected for this sample, although it was not possible to obtain a reconstruction below 14 Å resolution due to intrinsic flexibility (Supplementary Fig. 1e, f). Despite this, future work will seek to address whether the XPF–ERCC1 heterodimer and heterotetramer play distinct roles in DNA repair pathways.

XPF–ERCC1 cryo-EM structures described here reveal how binding a DNA-junction substrate is able to disengage the XPF helical domain from the XPF CM and release the heterodimeric 2×(HhH)_2_ domain. A role for the linker regions in enabling this release is likely. The released 2×(HhH)_2_ domain is then able to engage a minor groove in a dsDNA duplex adjacent to the DNA ds/ss junction and packs against the XPF nuclease–ERCC1 NLD dimer, as observed for structures of Mus81–Eme1 and *A. pernix* XPF. The repositioning of the dimeric 2×(HhH)_2_ domain has three consequences. First, it destabilises the autoinhibition interface with the XPF helical domain. Second, it exposes the dsDNA-binding surface of ERCC1 (HhH)_2_. Third, it enables the proper coupling of the ERCC1 ssDNA and dsDNA-binding functions by shortening the linker regions and forming a compact conformation with ERCC1–NLD–(HhH)_2_ domain contacts. The structures described here do not reveal the full basis for DNA-junction recognition or the extent of conformational flexing required to place the scissile bond proximal to the XPF catalytic centre. We speculate that the similarities between XPF HLM and the MDA5 helicase point to a concave surface that could engage the major groove of a DNA duplex within a DNA junction to promote movement of the ds-ssDNA discontinuity into the XPF catalytic site. Evidently further high-resolution structures are required with longer DNA substrates and recruitment partner complexes in order to fully understand how the scissile phosphodiester bond is presented to the XPF catalytic site and the extent of the conformational alterations required.

Whilst this paper was in preparation, the structure of a ds/ssDNA-bound TFIIH–XPA (PDB code: 6RO4) was published representing a 5′-NER pre-incision complex that can recruit XPF–ERCC1^37^. Superposition of the ERCC1 (HhH)_2_ domain–dsDNA complex onto the exposed DNA minor groove at the TFIIH–XPA–ds-ssDNA junction (Supplementary Fig. 8b) revealed a non-overlapping complementarity in DNA binding with XPA. ERCC1 engaged precisely the available DNA elements that were not engaged by XPA (Supplementary Fig. 8a). The resulting model predicts extensive interfaces between the XPF–ERCC1 and TFIIH–XPA–DNA with few steric clashes, many of which were within the flexible XPA loop region (residues 104–131). In this model, the dimeric 2×(HhH)_2_ domain lies adjacent to the TFIIH subunit XPB and DNA whilst the XPF nuclease–ERCC1 NLD dimer is positioned close to XPD, XPA and DNA. The highly basic and flexible RecA2 insert one (residues 345–377) is oriented to interact with either the extended XPA helix or dsDNA. Further structural studies are required to validate such a model.

Finally, there is a pressing need to explore chemical inhibition of XPF–ERCC1 to sensitise cancer cells to platinum-based therapeutics and reduce drug resistance mediated by XPF-ERCC1. Equally, XPF-ERCC1 inhibitors could target cancer cell vulnerabilities including XPF-FANCM synthetic lethality relevant to FANCM-deficient tumours^44^ and potentially other platinum-sensitive contexts^45^. The availability of an atomic structure for human XPF–ERCC1 described here will encourage efforts to develop new precision medicines as well as to overcome cancer chemoresistance^46^.

## Methods

### XPF–ERCC1 expression, purification and complex assembly

All reagents purchased from Sigma-Aldrich unless otherwise stated. A pFastBac Dual vector containing full length, wild type, human XPF (NCBI reference sequence: NM_005236.2) and ERCC1 (NCBI reference sequence: NM_001166049.2) cDNA was modified to include a C-terminal ERCC1 Twin-Strep-tag using restriction enzyme cloning. All primer sequences used in this study are shown in Supplementary Table 3. This plasmid was transformed into competent DH10_BAC_ *Escherichia coli* cells (Thermo-Fisher) and recombinant bacmid DNA purified. Recombinant baculoviruses expressing XPF and ERCC1 were generated using standard protocols^47^ (Oxford Expression Technologies). In short, 1 × 10^6^ SF21 cells (Thermo-Fisher) grown in SFIII media (Thermo-Fisher) and 10 μg/ml gentamycin (Life Technologies) were infected at a multiplicity of infection (MOI) of 2 and harvested after 72 h. Cell pellets were resuspended in extract buffer (20 mM HEPES pH 7.8, 150 mM NaCl, 1 mM tris(2-carboxyethyl)phosphine (TCEP), 10% glycerol, 2 mM MgCl_2_, 0.01% 3-((3-cholamidopropyl) dimethylammonio)-1-propanesulfonate (CHAPS), 0.25 tablet of EDTA-free protease-inhibitor cocktail per litre of culture, and 1 μl per 250 mL lysate BaseMuncher (Expedeon)) and lysed by sonication. The lysate was cleared of insoluble cell debris by centrifugation at 35,000*g* for 45 min and incubated with Strep-tactin resin (GE Healthcare) for 1 h at 4 °C. The resin was extensively washed with extract buffer minus protease inhibitors and BaseMuncher and incubated for 12 hours with Tobacco Etch Virus protease (supplier NEB). The eluate, containing XPF–ERCC1 was concentrated and loaded onto an anion-exchange column (HiTrap-Q, GE Healthcare) and XPF–ERCC1 containing fractions eluted using a gradient across 20 ml of extract buffer + 1 M NaCl before a final SEC step using a Superdex-200i column (GE Healthcare) in cryo buffer (20 mM HEPES pH 7.8, 150 mM NaCl, 1 mM TCEP, 0.01% CHAPS). Mutants were cloned using the Q5 site-directed mutagenesis kit (New-England Biotech) and were then expressed using the same protocol as described above for wild-type XPF–ERCC1.

### XPF–ERCC1 DNA complex assembly

DNA with a modified phosphorothioate backbone (SL_p_ DNA) was resuspended in DNA resuspension buffer (10 mM Tris, pH 7.8, 1 mM EDTA and 75 mM NaCl) and annealed to form a stem–loop structure. Purified XPF–ERCC1 was buffer exchanged into XPF–ERCC1 DNA cryo buffer (20 mM HEPES pH 7.8, 150 mM NaCl, 1 mM TCEP, 0.01% CHAPS, 5 mM CaCl_2_, 0.5 mM EDTA) and then incubated with SL_p_ DNA at a 1:2 protein:DNA molar ratio for 10 min at 4 °C followed by cross-linking with 0.05% (v/v) glutaraldehyde for 10 min at 4 °C. The cross-linking reaction was quenched by the addition of 1 mM Tris-HCl, pH 7.8 and the complex further purified via SEC using a Superdex 200i column.

Stem–loop sequence: CAGCG*C*T*U*G*G*TTTTTTTTTTTTTTTTTTTT*C*C*A*A*G*CGCTG, where the asterisk * represents a phosphorothioate backbone.

### XPF–ERCC1 cryo-EM grid preparation and data collection

For cryo-EM analysis, 4 μl of the purified XPF–ERCC1 heterodimer at 1.5 mg/ml was applied to both R1.2/1.3 400 mesh UltraFoil^®^ and QuantiFoil^®^ grids that had been previously glow discharged for 45 s at 42 mA. The grids were blotted for 4 s at 100% humidity and 4 °C and plunged into liquid ethane cooled by liquid nitrogen using a FEI Vitrobot MK IV. The grids were then loaded onto a Titan Krios transmission electron microscope operated at 300 kV (Thermo-Fisher). Images were collected in counting mode using a Gatan K2 Summit direct electron detector camera mounted behind a GIF Quantum energy filter operating in zero-loss mode. Exposures were 15 s, with a total dose of 63 e^−^/Å^2^ dose-fractionated into 40 frames with a calibrated pixel size of 1.38 Å. Images were recorded with a defocus of 1.5 µm to 4 µm. A total of 15,315 micrographs were collected from three separate data collection sessions.

### XPF–ERCC1 cryo-EM image processing

Movie frames were corrected for motion using MotionCor2^48^, and contrast transfer function was estimated using CTFfind4.1^49^ within Scipion1.2^50^. The total number of movies used for processing was 14,453. Two-hundred micrographs were selected from the first collection from which 82,412 particles were picked using Xmipp3^51^ semi-automated picking and extracted using RELION-3^52^. The particles were sorted using Xmipp3^51^ screen particles followed by three rounds of reference-free 2D classification in CryoSPARC-2^53^. A subset of six 2D classes were selected that represented different views of the molecule and used as templates for reference-based particle picking using Gautomatch^54^ on the full dataset. This approach yielded 396,106, 1,201,881 and 2,391,900 particles for data collection runs one, two and three, respectively. The particles were extracted and binned twofold using RELION-3^52^, sorted using Xmipp3^51^ to screen particles and then submitted for three rounds of reference-free 2D classification in CryoSPARC-2^53^. This reduced the particle numbers to 151,412, 390,007 and 1,074,111 particles for data collection runs one, two and three, respectively. Four initial models were generated using the ab initio reconstruction programme in CryoSPARC-2^53^ and were used as references for 3D classification using heterogeneous refinement in CryoSPARC-2^53^. Multiple rounds of heterogeneous refinement yielded 44,312, 126,492 and 390,712 particles in well-defined classes for data collection runs one, two and three respectively. All 561,516 particles from the three collections were re-extracted in an un-binned 200 ×200 pixel box using RELION-3^52^ and csparc2star and then merged. The data then underwent 3D classification without alignment in RELION-3^52^ to identify the most stable, high-resolution class. The two classes that displayed the highest-resolution features, comprising 405,339 particles, were refined to 4.1 Å resolution in CryoSPARC-2^53^ using non-uniform refinement. Per-particle motion correction was carried out using Bayesian polishing in RELION-3^52^. The shiny, polished particles were then refined to 4.0 Å resolution in CryoSPARC-2^53^ using non-uniform refinement.

**Table 4:** Cryo-EM statistics for XPF–ERCC1 structures and associated maps.

Data Collection and Processing	XPF–ERCC1	XPF–ERCC1_DNA_
EMDB ID	EMD-10337	EMD-10338
PDB ID	6SXA	6SXB
Magnification	36,232×	36,232×
Voltage (kV)	300	300
Electron exposure (e^−^/Å2)	63	63
Defocus range (μm)	1–4	1–3
Pixel size (Å)	1.38	1.38
Symmetry imposed	C1	C1
Initial particle images	3,989,887	3,432,565
Final particle images	405,339	199,022
Map resolution (Å)	3.6 (local refinement)	7.9 (globally refined)
	4.0 (globally refined)	7.1 (local refinement)
Map resolution range (Å)	3.4–8	5.5–14
*Refinement*		
Initial model	Cryo-SPARC ab initio	Cryo-SPARC ab initio
Model resolution (Å)	3.6–4	5.9–7.7
FSC threshold	0.5	0.5
Map sharpening B factor (Å^2^)	−168	−560
*Model composition*		
Non-hydrogen atoms	7218	7345
Protein residues	945	892
*B* factor (Å^2^)	163	530
*R.m.s. deviations*		
Bond lengths (Å)	0.004	0.23
Bond angles (°)	0.682	0.37
*Validation*		
MolProbity score	1.88	2.9
Clashscore	7.01	4
Poor rotamers (%)	0	0
*Ramachandran plot*		
Favoured (%)	91.7	99
Allowed (%)	8.4	1
Disallowed (%)	0	0

Inspection of the 4.0 Å resolution map rendered by local resolution in Chimera^55^ identified the dimeric XPF–ERCC1 2×(HhH)_2_ domain as the lowest resolution region of the map, suggesting some degree of mobility. A mask which excluded the low-resolution XPF–ERCC1 2×(HhH)_2_ hairpins was generated in Chimera^55^ and using the particle subtraction tool in CryoSPARC-2^53^ the portion of the particle images aligning to the hairpin density in the map was removed. Non-uniform local refinement in CryoSPARC-2^53^ was performed on the subtracted particles, re-aligning them to the masked reference volume, leading to a reconstruction at 3.6 Å resolution which excluded the hairpin portion of the 4.0 Å map.

All resolutions reported here were determined by Fourier shell correlation (at FSC = 0.143) based on the “gold-standard” protocol using a soft mask around the complex density^56^. To avoid over-masking, the masked maps were visually inspected to exclude the possibility of clipping. In addition, the occurrence of over-masking was monitored by inspecting the shapes of FSC curves. The two-half maps had their phases randomised beyond the resolution at which the no-mask FSC drops below the FSC = 0.143 criterion. The tight mask is applied to both half maps, and an FSC is calculated. This FSC is used along with the original FSC before phase randomisation to compute the corrected FSC. Local resolution was calculated using Blocres within CryoSPARC-2^53^. For visualisation, maps were sharpened by applying an automated local resolution weighted negative *B* factor using the local filtering function of CryoSPARC-2^53^.

### XPF–ERCC1 model building

Initially the crystal structures of the ERCC1 NLD (PDB code: 2A1I) and the tandem helix–hairpin–helix domains comprising XPF and ERCC1 chains (PDB code: 2A1J) were rigid body fitted into the locally filtered and sharpened map obtained at 4.0 Å resolution. Homology models were generated for the XPF RecA1 domain and rigid body fit into the map using the same procedure. Subsequently, the fitted domains were rebuilt manually using COOT^57^ optimising the fit where sidechain densities were evident prior to using FlexEM^38^ and real-space refinement as implemented in PHENIX^58^ whilst imposing secondary structural and geometric restraints to prevent overfitting (Table 4). The RecA2 and helical domains were built de novo and subjected to PHENIX^58^ real-space refinement. A further 6 cycles of rebuilding and refinement in COOT^57^ and PHENIX^58^ lead to a model containing 743 residues from XPF and 195 from ERCC1. Linkers regions connecting the XPF nuclease and ERCC1 NLD domains to their respective (HhH)_2_ domains were built manually into the map and the N-terminal portion of the XPF nuclease domain homology model was rebuilt in COOT^57^ to fit the map. The final atomic model was evaluated using MolProbity^59^ (Table 4). The location of patient mutations and sidechains referred to in the text are mapped onto the primary sequence, together with sequence conservation within XPF and ERCC1 homologues respectively (Supplementary Figs. 10 and 11).

### XPF–ERCC1–DNA complex cryo-EM grids and data collection

XPF–ERCC1–DNA complex was concentrated to 1.3 mg/ml and applied to Quantifoil R1.2/1.3 300 mesh copper grids. The freezing and imaging conditions used were the same as for the DNA-free XPF–ERCC1 complex described above. A total of 8965 movies were collected from a single data collection using the same electron microscope and detector as described above.

### XPF–ERCC1–DNA complex cryo-EM image processing

Motion correction and CTF estimation was performed as previously described for the XPF–ERCC1 data collections. Totally, 7982 micrographs were manually selected for processing. Particle picking was carried out as described for the XPF–ERCC1 data collections. 3,432,565 particles were extracted and sorted using Xmipp3^51^ screen particles and then submitted for six rounds of reference-free 2D classification in CryoSPARC-2^53^. A total of 688,821 particles were used to generate 4 ab initio reconstructions which were then used as references for 3D classification using heterogeneous refinement in CryoSPARC-2^53^. Multiple rounds of heterogeneous refinement were carried out yielding one well-ordered reconstruction comprising 199,022 particle images (Table 4). This class was refined to 7.7 Å resolution using non-uniform refinement in CryoSPARC-2^53^. A mask was generated using UCSF Chimera^55^ that excluded both the DNA and hairpin domain density which was used to carry out masked refinement improving the resolution of the sub-volume to 5.9 Å (Table 4).

### XPF–ERCC1–DNA complex model building

Individual domains of XPF–ERCC1 were taken from the DNA-free structure and fitted into the DNA-bound cryo-EM map density as rigid bodies using the UCSF Chimera^55^ fit-in-map tool. The homodimeric *A. pernix* XPF (PDB:2BGW) bound to dsDNA through its (HhH)_2_ hairpins was fitted into the DNA-bound map density and the subsequent position of the DNA-bound *A. pernix* hairpins used as a reference to align the human hairpin domain using MatchMaker in UCSF Chimera^55^. The DNA from the *A. pernix* structure was reduced to a 10 base-pair duplex and modelled into the map whilst preserving the hairpin domain–DNA contacts. The sequence conservation of the functional human ERCC1 and *A. pernix* (HhH)_2_ domains is high: 25.5% identical and 69.1% similar residues. The ds-RNA bound structure of MDA5 (PDB: 4GL2) was placed into the DNA-bound map density as a guide to place the helical domain of XPF by inspecting the position of the homologous domain in MDA5.

### XPF–ERCC1–DNA–TFIIH–XPA complex modelling

The XPF–ERCC1–DNA structure was aligned to the TFIIH–DNA–XPA structure (PDB code: 6RO4) through structural super-imposition in UCSF Chimera^55^ and alignment with the two DNA strands of a single duplex from each structure. The ds/ss DNA junction was defined by the high-resolution DNA structure in the TFIIH–XPA complex and demarcated by the position of the XPA β-hairpin.

### XPF–ERCC1 cross-linking mass spectrometry

All chemicals were purchased from Sigma-Aldrich unless otherwise stated. A total of 100 µg XPF–ERCC1 heterodimer at a concentration of 1 mg/ml in 20 mM HEPES, pH 7.8, 10% Glycerol, 0.01% CHAPS, 150 mM NaCl, 1 mM TCEP, 0.5 mM EDTA was cross-linked using 1 mM disuccinimidyl sulfoxide (DSSO) (Thermo-Fisher) with mild shaking for 30 min at 37 °C. The reaction was quenched using a final concentration of 50 mM ammonium bicarbonate for a further 20 min at 37 °C. To remove potential aggregates, gradient ultracentrifugation was employed using a 5–30% glycerol gradient in 20 mM Hepes, 150 mM NaCl, mixed using a Gradient Master (BioComp), and centrifuged for 16 h at 4 °C at 200,000×*g* using a SW 55 Ti Rotor (Beckman Coulter)^60^. Totally, 100 µL fractions were collected and silver stained to identify fractions containing cross-linked non-aggregated XPF–ERCC1. Fractions containing cross-linked proteins were then pooled and buffer exchanged into 8 M urea using a Vivaspin 500, 30,000 molecular weight cut off (MWCO) PES filter (Sartorius, VS0122). Cysteine reduction was carried out using 2.5 mM TCEP for 30 min at 37 °C and alkylated in the dark using 5 mM iodoacetamide at room temperature. The urea was then buffer exchanged for 50 mM ammonium bicarbonate and proteins were proteolysed using trypsin (Promega) at 1:50 w/w trypsin:protein overnight at 37 °C. The solution was acidified using 2% formic acid and peptides were the spun through the MWCO filter and desalted using in-house built STAGE tips made using Empore SPE C18 discs (3 M, 66883-U). The eluent was then dried to completion. Peptides were reconstituted in 0.1% trifluoroacetic acid (TFA) and chromatographically resolved using an Ultimate 3000 RSLCnano (Dionex) HPLC. Peptides were first loaded onto an Acclaim PepMap 100 C18, 3 µm particle size, 100 Å pore size, 20 mm × 75 µm ID (Thermo Scientific, 164535) trap column using a loading buffer (2% acetonitrile (MeCN) and 0.05% TFA in 97.05 % H_2_O) with a flow rate of 7 µL/min. Chromatographic separation was achieved using an EASY-Spray column, PepMap C18, 2 µm particles, 100 Å pore size, 500 mm × 75 µm ID (Thermo Scientific, ES803). The gradient utilised a flow of 0.3 µl/min, starting at 98% mobile A (0.1% formic acid, 5% dimethyl sulfoxide (DMSO) in H_2_O) and 2% mobile B (0.1% formic acid, 75% MeCN, 5% DMSO and 19.9% H_2_O). After 6 min, mobile B was increased to 30% over 69 min, to 45% over 30 min, further increased to 90% in 16 min and held for 4 min. Finally, Mobile B was reduced back to 5% over 1 min for the rest of the acquisition. Data were acquired in real time over 140 min using an Orbitrap Fusion Lumos Tribrid mass spectrometer in positive, top speed mode with a cycle time of 5 s. The chromatogram (MS1) was captured using 60,000 resolution, a scan range of 375–1500 with a 50 ms maximum injection time, and 4e5 AGC target. Dynamic exclusion with repeat count 2, exclusion duration of 30 s, 20 ppm tolerance window was used, along with isotope exclusion, a minimum intensity exclusion of 2e4, charge state inclusion of 3–8 ions and peptide mono isotopic precursor selection. Precursors within a 1.6 *m*/*z* isolation window were then fragmented using 25% normalised CID, 100 ms maximum injection time and 5e4 AGC target. Scans were recorded using 30,000 resolution in centroid mode, with a scan range of 120–2000 *m*/*z*. Spectra containing peaks with a mass difference of 31.9721 Da were further fragmented with a 30% normalised higher collision induced dissociation, using a 2 *m*/*z* isolation window, 150 ms maximum injection time and 2e4 AGC target. Four scans were recorded using an ion trap detection in rapid mode starting at 120 *m*/*z*.

### XL-MS data analysis

Data processing were carried out using Proteome Discoverer Version 2.4 (Thermo Scientific) with the XlinkX^61^ node where the minimum XlinkX score was set to 63. The acquisition strategy was set to MS2_MS3 mode. The database comprised solely of the specific XPF and ERCC1 sequences. Trypsin was selected as the proteolytic enzyme allowing up to two missed cleavages with a minimal peptide length of five residues. Masses considered were in the range of 300–10000 Da. The precursor mass tolerance, FTMS fragment mass tolerance, and ITMS Fragment Mass Tolerance were set to 10 ppm, 20 ppm and 0.6 Da, respectively. A static carbamidomethyl (+57.021 Da) modification was utilised for cysteine residues, with additional dynamic modifications considered including; amidated and hydrolysed DSSO (+142.050 and +176.014 Da, respectively) on lysine serine and threonine residues, oxidation (+15.995 Da) on methionine residues, and protein N-terminal acetylation (+42.011 Da). The FDR threshold was set to one with the strategy set to simple. The list of reported cross-linked spectral matches were manually examined and cross-links with spectra that did not contain acceptable b and y ion coverage were excluded. We note that this method requires accessible lysine sidechains therefore predominantly hydrophobic interfaces, such as the RecA1–nuclease, did not return any cross-links^62^. A number of cross-links were observed that exceed the permitted the Cα–Cα cut-off distance of 30 Å.

### XPF–ERCC1–SLX4^NTD^ complex assembly

cDNA encoding the SLX4^NTD^ (residues 1–758) (NCBI reference sequence: NM_032444) was shuttled into a pGEX-1 vector (Sigma). Recombinant baculoviruses expressing the SLX4^NTD^ were generated as previously described and used to infect 1 × 10^6^ SF21 cells (Thermo-Fisher) grown in SFIII media (Thermo-Fisher) and 10 μg/ml gentamycin (Life Technologies) at an MOI of 0.5. These cells were co-infected with XPF–ERCC1 expressing baculovirus at an MOI of 2. Cells were pelleted after 72 h and protein extracted as previously described for XPF–ERCC1. Following Strep-tactin affinity purification, the complex was purified using anion-exchange (HiTrap-Q, GE Healthcare) using a gradient of 150 mM NaCl to 500 mM NaCl over 20 ml of extract buffer minus protease inhibitors and BaseMuncher. This separated the SLX4^NTD^–XPF–ERCC1 complex from unbound XPF–ERCC1. Fractions containing the SLX4^NTD^–XPF–ERCC1 complex were pooled and concentrated prior to a final SEC step using a Superose-6 increase column equilibrated in extract buffer minus protease inhibitors and BaseMuncher (GE Healthcare). Fractions containing both XPF and SLX4^NTD^ were identified via Western blot.

### Real-time fluorescence incision assay

Fluorescently labelled stem–loop (SL_F_) DNA substrates, containing a 5′ 6-FAM fluorophore and 3′-BHQ1 quench, were purified by SEC (Superdex-200i, GE Healthcare) in assay buffer (5 mM HEPES, 10% glycerol, 0.5 mM DTT, 1 mM MnCl_2_ and 40 mM NaCl. The purified substrates were then annealed by heating to 95 °C for 1 min followed by cooling to 4 °C and dispensed into the assay plate. Reactions were carried out in 384-well black, flat-bottomed microtitre plates (Corning 3854). Purified XPF–ERCC1 was buffer exchanged into assay buffer and 5 nM added to each in a total volume of 20 µl to initiate the endonuclease reaction. Fluorescence measurements were carried out using the CLARIOstar plate reader (BMG Labtech) using an excitation wavelength of 483 nm and an emission wavelength of 525 nm. Sixty readings were collected at 30-s intervals and the linear response range for each substrate was used to determine the change in fluorescence per unit time. Kinetic parameters were calculated using the Michaelis–Menten equation. Experimental product release was quantified by plotting the relative fluorescence units produced by known amounts of the cleavage products against their concentration to generate a standard curve.

SL_F_ sequence: 6-FAM-5′-CAGCGCTUGGTTTTTTTTTTTTTTTTTTTTCCAAGCGCTG-3′-BHQ1.

Cleavage product #1: 6-FAM-5′-CAGCGCTC 3′.

Cleavage product #2: 5′-GGTTTTTTTTTTTTTTTTTTTTCCGAGCGCTG-3′-BHQ1.

### Reporting summary

Further information on research design is available in the Nature Research Reporting Summary linked to this article.

## Supplementary information

Supplementary Information

Description of Additional Supplementary Files

Supplementary Movie 1

Supplementary Movie 2

Supplementary Movie 3

Supplementary Movie 4

Supplementary Movie 5

Supplementary Movie 6

Supplementary Movie 7

Supplementary Movie 8

Supplementary Movie 9

Supplementary Movie 10

Reporting Summary

## Data Availability

The coordinates for the DNA-free and DNA-bound XPF–ERCC1 complex are available in the PDB with codes 6SXA [https://www.ebi.ac.uk/pdbe/entry/pdb/6sxb] and 6SXB [https://www.ebi.ac.uk/pdbe/entry/pdb/6sxb] and the cryo-EM maps are available in EMDB with codes EMD-10337 [https://www.ebi.ac.uk/pdbe/entry/emdb/EMD-10337] and EMD-10338 [https://www.ebi.ac.uk/pdbe/entry/emdb/EMD-10337]. The source data underlying Figs. 1b, 5b Supplementary Figs. 1b, 9d, e are provided as a Source Data file. Other data that support the findings of this study are available from the corresponding author upon request.

## Source Data

Source Data

## References

[1] Dehe PM, Gaillard PH (2017). Control of structure-specific endonucleases to maintain genome stability. Nat. Rev. Mol. Cell Biol..

[2] Ciccia A, McDonald N, West SC (2008). Structural and functional relationships of the XPF/MUS81 family of proteins. Annu Rev. Biochem..

[3] Faridounnia M, Folkers GE, Boelens R (2018). Function and interactions of ERCC1-XPF in DNA damage response. Molecules.

[4] Marteijn JA, Lans H, Vermeulen W, Hoeijmakers JHJ (2014). Understanding nucleotide excision repair and its roles in cancer and ageing. Nat. Rev. Mol. Cell Biol..

[5] Klein Douwel D, Hoogenboom WS, Boonen RACM, Knipscheer P (2017). Recruitment and positioning determine the specific role of the XPF‐ERCC1 endonuclease in interstrand crosslink repair. EMBO J..

[6] Wyatt HDM, Laister RC, Martin SR, Arrowsmith CH, West SC (2017). The SMX DNA repair tri-nuclease. Mol. Cell.

[7] Wu Y, Mitchell TR, Zhu XD (2008). Human XPF controls TRF2 and telomere length maintenance through distinctive mechanisms. Mech. Ageing Dev..

[8] Woodrick J (2017). A new sub‐pathway of long‐patch base excision repair involving 5′ gap formation. EMBO J..

[9] Al-Minawi AZ, Saleh-Gohari N, Helleday T (2008). The ERCC1/XPF endonuclease is required for efficient single-strand annealing and gene conversion in mammalian cells. Nucleic Acids Res..

[10] Ahmad A (2008). ERCC1-XPF endonuclease facilitates DNA double-strand break repair. Mol. Cell Biol..

[11] Bogliolo M (2013). Mutations in ERCC4, encoding the DNA-repair endonuclease XPF, cause Fanconi anemia. Am. J. Hum. Genet..

[12] Jaspers NGJ (2007). First reported patient with human ERCC1 deficiency has cerebro-oculo-facio-skeletal syndrome with a mild defect in nucleotide excision repair and severe developmental failure. Am. J. Hum. Genet..

[13] Kashiyama K (2013). Malfunction of nuclease ERCC1-XPF results in diverse clinical manifestations and causes Cockayne syndrome, xeroderma pigmentosum, and Fanconi anemia. Am. J. Hum. Genet..

[14] Niedernhofer LJ (2006). A new progeroid syndrome reveals that genotoxic stress suppresses the somatotroph axis. Nature.

[15] Sijbers AM (1996). Xeroderma pigmentosum group F caused by a defect in a structure-specific DNA repair endonuclease. Cell.

[16] Fairman-Williams ME, Guenther UP, Jankowsky E (2010). SF1 and SF2 helicases: family matters. Curr. Opin. Struct. Biol..

[17] Sgouros J, Gaillard PH, Wood RD (1999). A relationship betweena DNA-repair/recombination nuclease family and archaeal helicases. Trends Biochem. Sci..

[18] Gaillard PH, Wood RD (2001). Activity of individual ERCC1 and XPF subunits in DNA nucleotide excision repair. Nucleic Acids Res..

[19] Klein Douwel, D., Hoogenboom, W. S., Boonen, R. A. & Knipscheer, P. Recruitment and positioning determine the specific role of the XPF-ERCC1 endonuclease in interstrand crosslink repair. *Embo J*. **37**, 2034–2046 (2017). 10.15252/embj.201695223 PMC5510004 28292785

[20] Bowles M (2012). Fluorescence-based incision assay for human XPF-ERCC1 activity identifies important elements of DNA junction recognition. Nucleic Acids Res..

[21] Enzlin JH, Scharer OD (2002). The active site of the DNA repair endonuclease XPF-ERCC1 forms a highly conserved nuclease motif. EMBO J..

[22] Orelli B (2010). The XPA-binding domain of ERCC1 is required for nucleotide excision repair but not other DNA repair pathways. J. Biol. Chem..

[23] Yan L, Wu H, Li X, Gao N, Chen Z (2019). Structures of the ISWI-nucleosome complex reveal a conserved mechanism of chromatin remodeling. Nat. Struct. Mol. Biol..

[24] Eustermann S (2018). Structural basis for ATP-dependent chromatin remodelling by the INO80 complex. Nature.

[25] Newman M (2005). Structure of an XPF endonuclease with and without DNA suggests a model for substrate recognition. EMBO J..

[26] Tsodikov OV, Enzlin JH, Scharer OD, Ellenberger T (2005). Crystal structure and DNA binding functions of ERCC1, a subunit of the DNA structure-specific endonuclease XPF-ERCC1. Proc. Natl Acad. Sci. USA.

[27] Gwon GH (2014). Crystal structures of the structure-selective nuclease Mus81-Eme1 bound to flap DNA substrates. EMBO J..

[28] Coulthard R (2013). Architecture and DNA recognition elements of the Fanconi anemia FANCM-FAAP24 complex. Structure.

[29] Tripsianes K (2005). The structure of the human ERCC1/XPF interaction domains reveals a complementary role for the two proteins in nucleotide excision repair. Structure.

[30] Tsodikov OV (2007). Structural basis for the recruitment of ERCC1-XPF to nucleotide excision repair complexes by XPA. EMBO J..

[31] Marín M (2019). Functional comparison of XPF missense mutations associated to multiple DNA repair disorders. Genes.

[32] Matsumura Y, Nishigori C, Yagi T, Imamura S, Takebe H (1998). Characterization of molecular defects in xeroderma pigmentosum group F in relation to its clinically mild symptoms. Hum. Mol. Genet..

[33] Sijbers AM (1998). Homozygous R788W point mutation in the XPF gene of a patient with xeroderma pigmentosum and late-onset neurologic disease. J. Invest. Dermatol..

[34] Klein Douwel D (2014). XPF-ERCC1 acts in Unhooking DNA interstrand crosslinks in cooperation with FANCD2 and FANCP/SLX4. Mol. Cell.

[35] Hodskinson MR (2014). Mouse SLX4 is a tumor suppressor that stimulates the activity of the nuclease XPF-ERCC1 in DNA crosslink repair. Mol. Cell.

[36] Hoogenboom WS, Boonen R, Knipscheer P (2019). The role of SLX4 and its associated nucleases in DNA interstrand crosslink repair. Nucleic Acids Res..

[37] Kokic G (2019). Structural basis of TFIIH activation for nucleotide excision repair. Nat. Commun..

[38] Topf M (2008). Protein structure fitting and refinement guided by cryo-EM density. Structure.

[39] Das D (2017). Single-stranded DNA binding by the helix-hairpin-helix domain of XPF protein contributes to the substrate specificity of the ERCC1-XPF protein complex. J. Biol. Chem..

[40] Wu B (2013). Structural basis for dsRNA recognition, filament formation, and antiviral signal activation by MDA5. Cell.

[41] Holm Liisa (2019). Benchmarking fold detection by DaliLite v.5. Bioinformatics.

[42] Yu Q, Qu K, Modis Y (2018). Cryo-EM structures of MDA5-dsRNA filaments at different stages of ATP hydrolysis. Mol. Cell.

[43] Abdullah Ummi B, McGouran Joanna F, Brolih Sanja, Ptchelkine Denis, El‐Sagheer Afaf H, Brown Tom, McHugh Peter J (2017). RPA activates the XPF ‐ ERCC 1 endonuclease to initiate processing of DNA interstrand crosslinks. The EMBO Journal.

[44] Li S (2019). ERCC1/XPF is important for repair of DNA double-strand breaks containing secondary structures. iScience.

[45] Mesquita KA (2019). ERCC1-XPF deficiency is a predictor of olaparib induced synthetic lethality and platinum sensitivity in epithelial ovarian cancers. Gynecol. Oncol..

[46] McNeil EM, Melton DW (2012). DNA repair endonuclease ERCC1-XPF as a novel therapeutic target to overcome chemoresistance in cancer therapy. Nucleic Acids Res..

[47] Kost TA, Condreay JP (1999). Recombinant baculoviruses as expression vectors for insect and mammalian cells. Curr. Opin. Biotechnol..

[48] Zheng SQ (2017). MotionCor2: anisotropic correction of beam-induced motion for improved cryo-electron microscopy. Nat. Methods.

[49] Rohou A, Grigorieff N (2015). CTFFIND4: fast and accurate defocus estimation from electron micrographs. J. Struct. Biol..

[50] de la Rosa-Trevin JM (2016). Scipion: a software framework toward integration, reproducibility and validation in 3D electron microscopy. J. Struct. Biol..

[51] de la Rosa-Trevin JM (2013). Xmipp 3.0: an improved software suite for image processing in electron microscopy. J. Struct. Biol..

[52] Zivanov J (2018). New tools for automated high-resolution cryo-EM structure determination in RELION-3. Elife.

[53] Punjani A, Rubinstein JL, Fleet DJ, Brubaker MA (2017). cryoSPARC: algorithms for rapid unsupervised cryo-EM structure determination. Nat. Methods.

[54] Zhang, K. MRC, LMB. www.mrc-lmb.cam.ac.uk/kzhang/.

[55] Pettersen EF (2004). UCSF chimera—a visualization system for exploratory research and analysis. J. Comput. Chem..

[56] Rosenthal PB, Henderson R (2003). Optimal determination of particle orientation, absolute hand, and contrast loss in single-particle electron cryomicroscopy. J. Mol. Biol..

[57] Brown A (2015). Tools for macromolecular model building and refinement into electron cryo-microscopy reconstructions. Acta Crystallogr D Biol. Crystallogr..

[58] Afonine PV (2018). Real-space refinement in PHENIX for cryo-EM and crystallography. Acta Crystallogr. D Struct. Biol..

[59] Williams CJ (2018). MolProbity: more and better reference data for improved all-atom structure validation. Protein Sci..

[60] Kao A (2011). Development of a novel cross-linking strategy for fast and accurate identification of cross-linked peptides of protein complexes. Mol. Cell Proteom..

[61] de Graaf SC, Klykov O, van den Toorn H, Scheltema RA (2019). Cross-ID: analysis and visualization of complex XL-MS-driven protein interaction networks. J. Proteome Res..

[62] O’Reilly FJ, Rappsilber J (2018). Cross-linking mass spectrometry: methods and applications in structural, molecular and systems biology. Nat. Struct. Mol. Biol..

