# Supplementary material for: Cryo-EM structures of the XPF-ERCC1 endonuclease reveal how DNA-junction engagement disrupts an auto-inhibited conformation
Source: Nat Commun. 2020 Feb 28;11:1120. doi: 10.1038/s41467-020-14856-2 (PMC7048804; doi:10.1038/s41467-020-14856-2)
Supplement: Supplementary file 1 — Supplementary Information [file 41467_2020_14856_MOESM1_ESM.pdf]

Supplementary Information for:

Cryo-EM structures of the XPF-ERCC1 endonuclease reveal how DNA-junction engagement disrupts an auto-inhibited conformation

Morgan Jones et al.

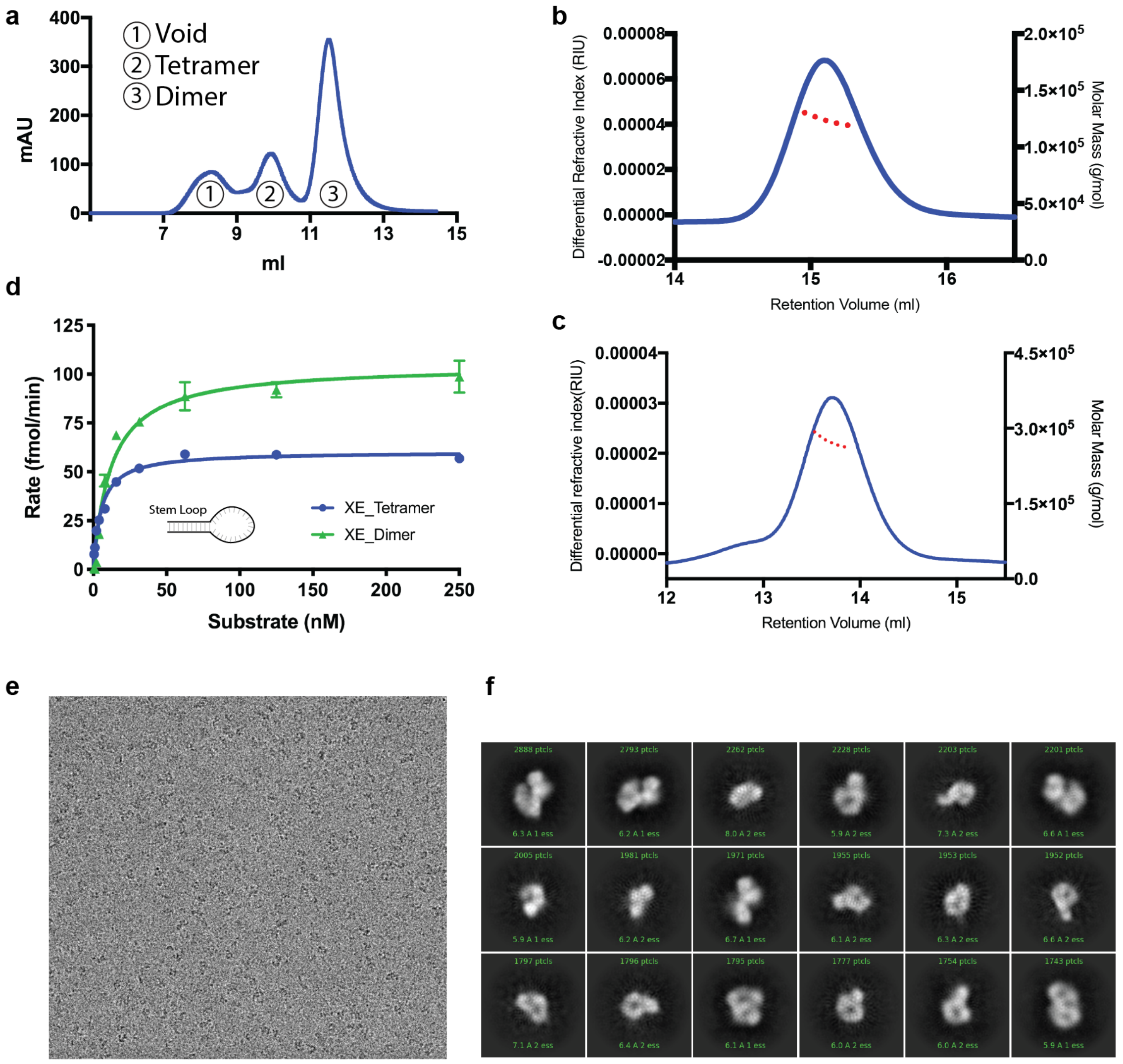

Supplementary Figure 1. XPF-ERCC1 purification characterisation. Recombinant full-length human XPF-ERCC1 heterodimer was produced using the baculovirus expression system in SF21 insect cells[48]. The sample was purified to homogeneity using a dual-Strep affinity-tag, anion exchange and size-exclusion chromatography. **a** Superdex-200 increase (SD200i) column trace for the XPF-ERCC1 complex purified by affinity and anion exchange chromatography. Peak 1 at 7.5ml is aggregated protein, peak 2 at 10ml is a heterotetrameric form (XPF-ERCC1)$_2$, peak 3 at 11.5ml is the predominant heterodimeric XPF-ERCC1 state. Samples from the heterodimer fraction were used for structural studies. **b-c** SEC-MALLS Superose-6 column traces with the estimated molecular mass overlaid in red. **b** XPF-ERCC1 heterodimer with molecular mass of 128kDa, approximately 9kDa less than the full-length molecular weight expected most likely due to the proteolytic cleavage of the amino-terminus of ERCC1 (~ 1-95 amino acids). **c** (XPF-ERCC1)$_2$ heterotetramer with molecular mass of 290kDa. **d** Michaelis-Menten kinetics plot of rate vs substrate concentration for the XPF-

ERCC1 heterodimer (green) and $(XPF\text{-}ERCC1)_2$ heterotetramer (blue). XPF-ERCC1 V_{max} – 104.6 fmol/min, K_m – 24.2 fmol, k_{cat} – 10.5 min^{-1}. $(XPF\text{-}ERCC1)_2$ V_{max} – 60.1 fmol/min, K_m – 21.0 fmol, k_{cat} -12.1 min^{-1}. Error bars indicate standard deviation (n=3). **e** Cryo-EM micrograph of $(XPF\text{-}ERCC1)_2$ collected using a Titan Krios, 1.09 Å/pixel using a Falcon III detector in counting mode. **f** 2D class averages of $(XPF\text{-}ERCC1)_2$ highlighting the flexible nature of the sample, precluding a high-resolution reconstruction.

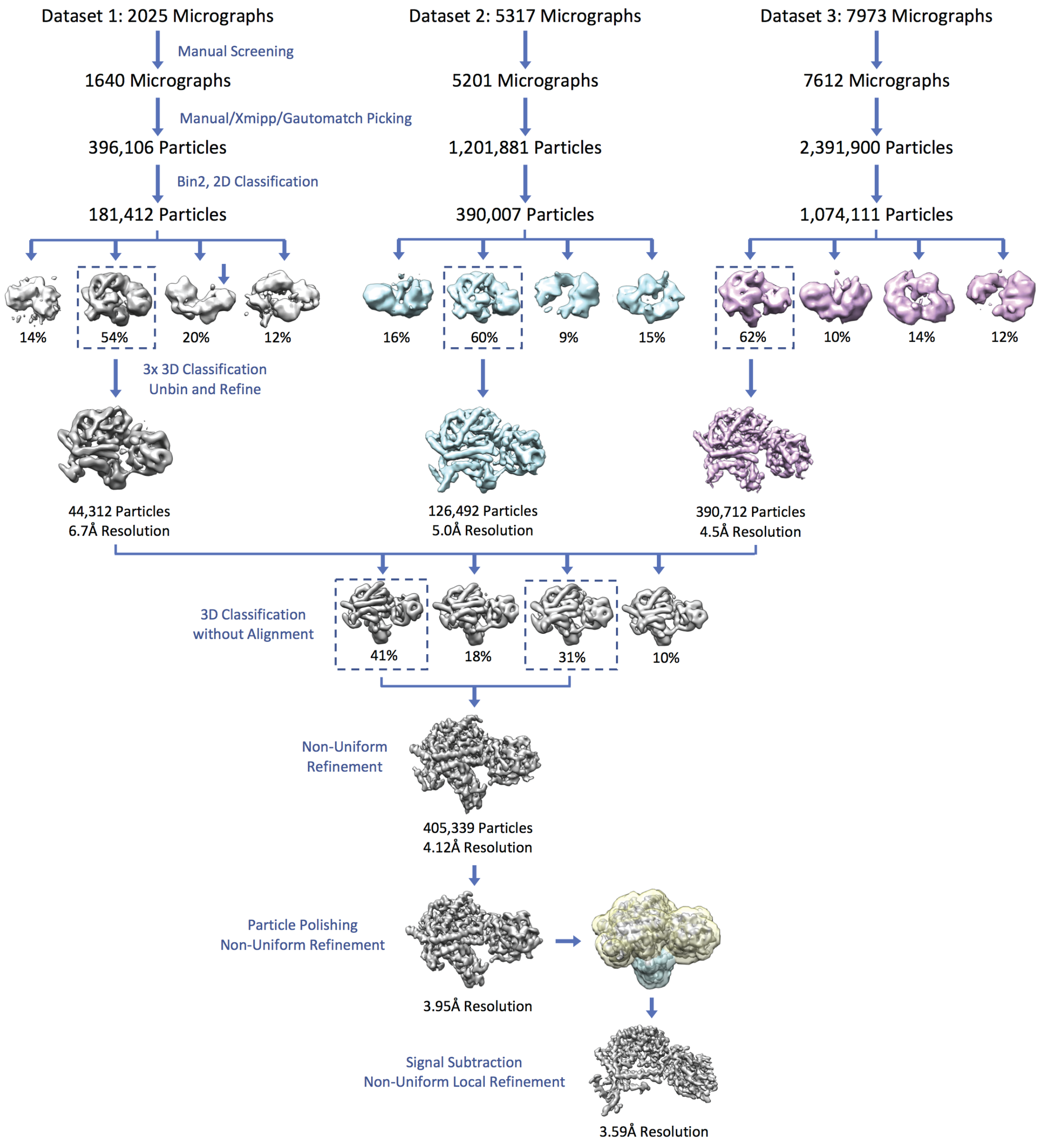

Supplementary Figure 2. XPF-ERCC1 cryo-EM processing workflow. Datasets one, two and three were initially processed independently. Micrographs were manually screened for crystalline ice and then particle images extracted following picking. After multiple rounds of 2D and 3D classification in CRYOSPARC-2[49] the data were merged and subjected to 3D classification without alignment in RELION-3[50]. Particles belonging to the two highest resolution classes were selected and refined followed by polishing in RELION-3[50]. The data were refined to 3.95 Å in CRYOSPARC-2[49]. Density corresponding to the dimeric $(HhH)_2$ hairpin domain was subtracted from the particle images and the remaining density was locally refined to 3.6 Å resolution.

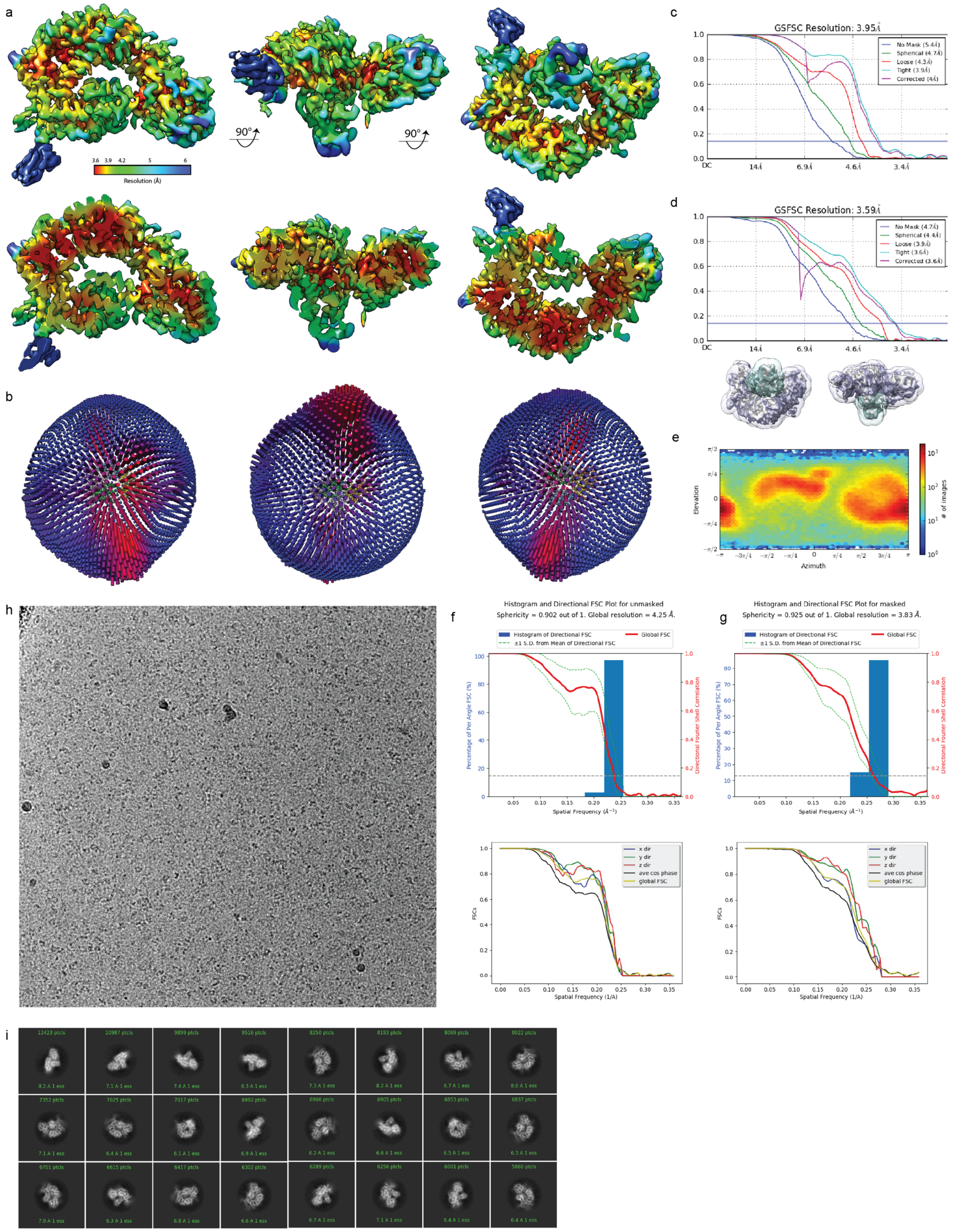

Supplementary Figure 3. DNA-free XPF-ERCC1 reconstruction quality. a XPF-ERCC1 composite map. The XPF RecA1, RecA2, helical and nuclease domains and ERCC1 NLD were all derived from local refinement at 3.6 Å omitting the 2x(HhH)$_2$ domain. The 2x(HhH)$_2$ domain structure was obtained from global refinement at 4.0 Å resolution. Top, three views of the composite map rendered based on local resolution. Bottom, same views cut through

density. **b** Distribution of particle images contributing to global reconstruction in RELION-2[51] **c** Fourier-shell correlation (FSC) curve for global refinement at 4.0 Å resolution. FSC = 0.143. **d** FSC curve for local refinement at 3.6 Å resolution. FSC = 0.143. displayed in 3D.Two views of XPF-ERCC1 highlighting the mask (in green) used for signal subtraction of the 2x$(HhH)_2$ domain **e** Distribution of particle images contributing to global reconstruction in CRYOSPARC-2[49] displayed in 2D. **f-g** 3D-FSC analysis for the global (unmasked) and locally (masked) refined XPF-ERCC1 structures. **f** Global 3D-FSC analysis **g** Local 3D-FSC analysis **h** Motion-corrected cryo-EM micrograph of the XPF-ERCC1 heterodimer. 1.38 Å/pixel collected using a K2 detector on a Titan Krios microscope. **i** Representative selection of cryo-EM 2D class-averages of XPF-ERCC1 calculated using CRYOSPARC-2[49] after final 3D classification. A mask with a diameter of 140 Å was applied.

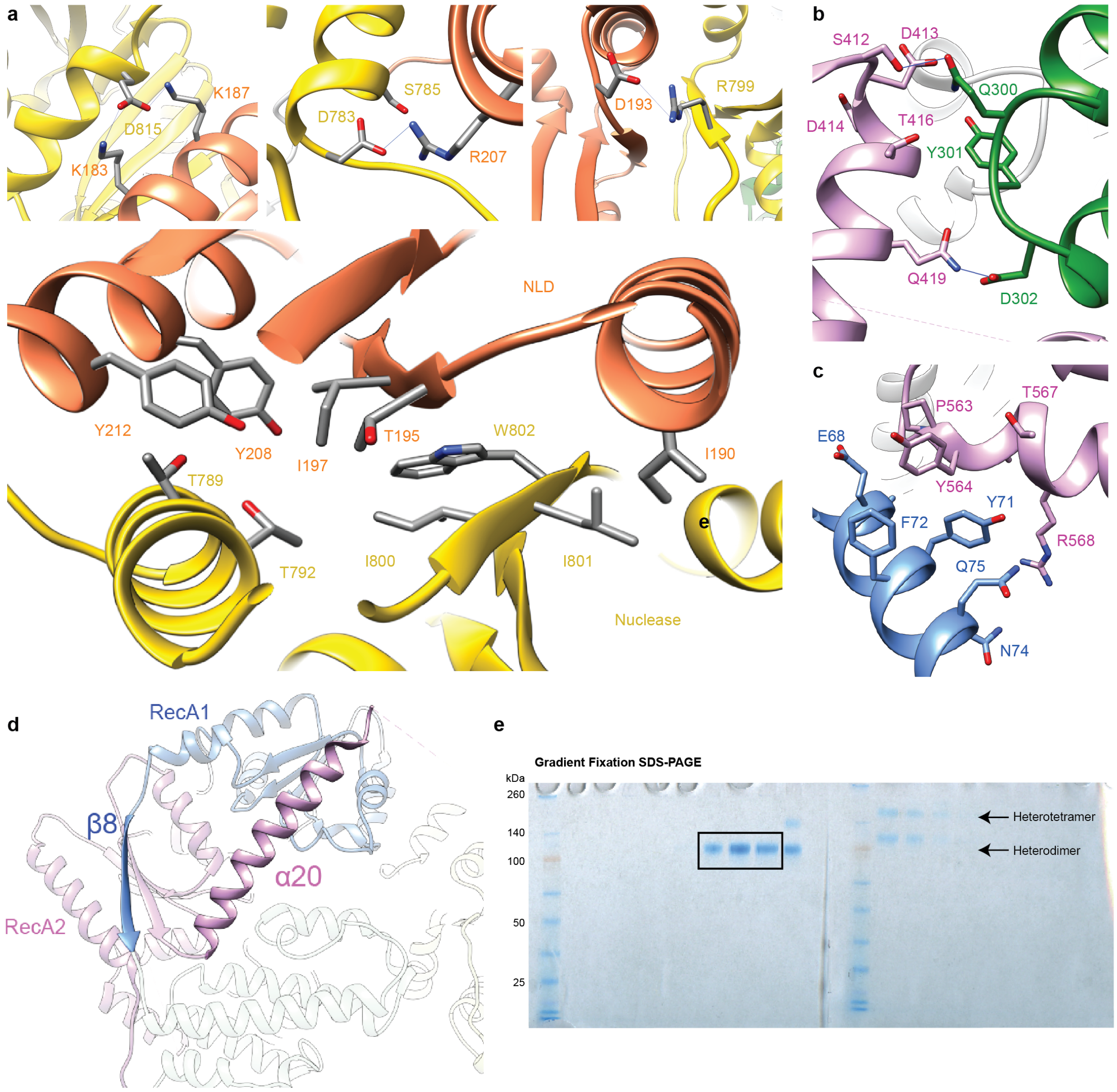

Supplementary Figure 4. Additional inter-domain contacts and XLMS data. Residues displayed as sticks and coloured by heteroatom, grey – C, blue – N, red – O. Proposed hydrogen bonds indicated by blue lines between residues. **a** Multiple panels showing views of XPF nuclease (gold) – ERCC1 NLD (coral) interface. Top three images show the salt bridges formed at the periphery of the interface. Bottom, hydrophobic residues at the core of the interface. **b** RecA2 (purple) – helical domain (green) interface highlighting the hydrogen bonds formed between S412 and Q300 and Q419 and D302. **c** RecA1 (blue) – RecA2 (purple) interface highlighting ring stacking between Y71 and Y654. **d** RecA1/A2 domain dimer. Key intertwined secondary structure elements highlighted. **e** Reducing SDS-PAGE gel of XPF-ERCC1 following gradient fixation using DSSO crosslinker. Fractions containing the heterodimer alone (boxed) were used for mass-spectrometry analysis.

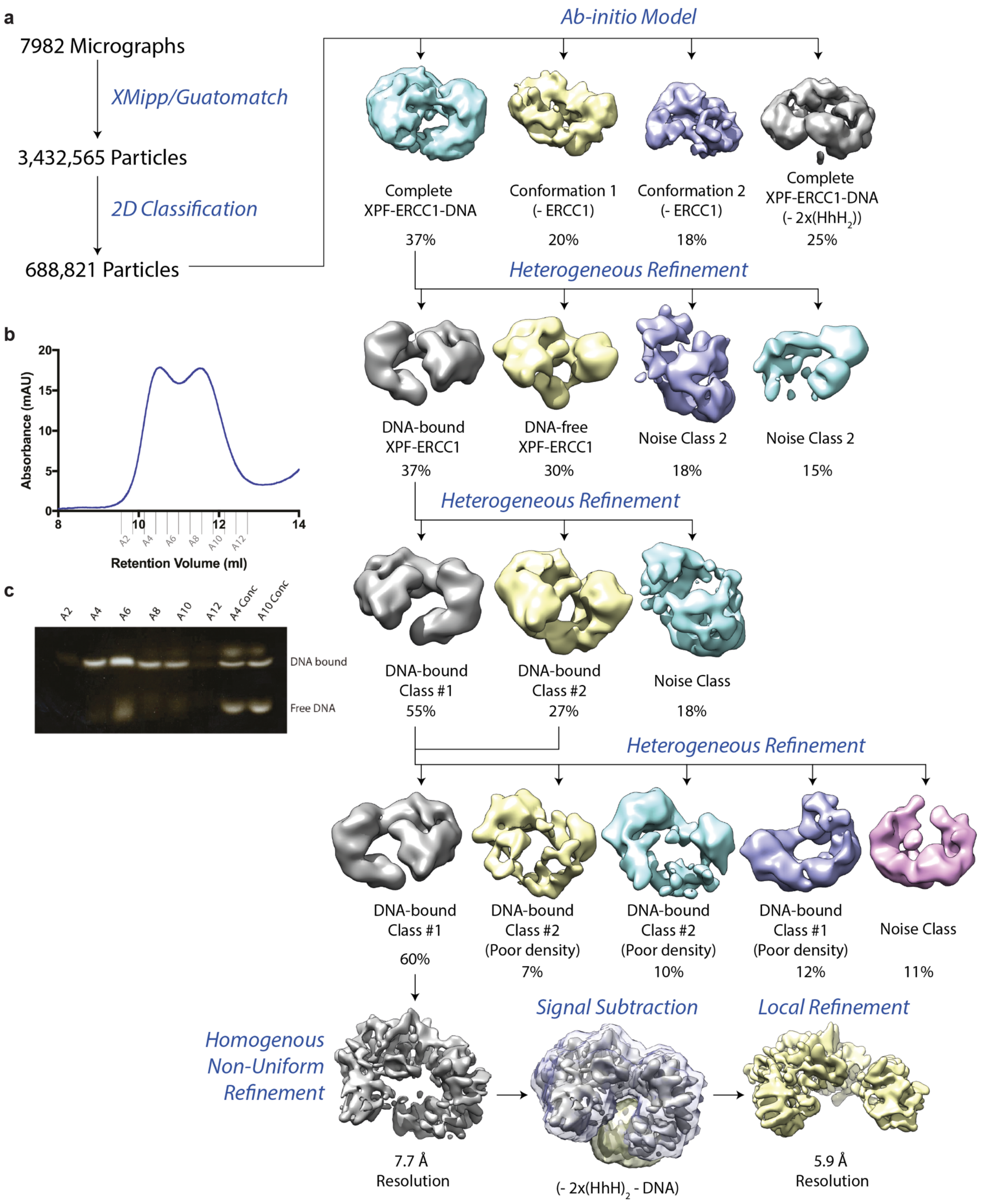

Supplementary Figure 5. XPF-ERCC1-DNA cryo-EM sample preparation and processing workflow. a Micrographs were manually screened for crystalline ice and then particle images extracted following picking. After 5 rounds of 2D classification the particle images displaying high-resolution features were classified in 3D using heterogeneous refinement in CRYOSPARC-2[49] using multiple 3D references generated by *ab-initio* reconstruction. The 3D class with the volume sufficient to accommodate all the domains of XPF-ERCC1 was selected and classified a further 3 times using the same routine. 3D classification was used to discard

DNA-free XPF-ERCC1 and classes in which the density was not continuous suggesting either an incomplete complex or poor alignment. After 3 rounds of 3D classification each class was subjected to homogenous non-uniform refinement. The only 3D class that refined to below 10 Å resolution was identified as intact XPF-ERCC1 bound to the dsDNA element of the stem loop. This class, consisting of 100,307 particle images was refined to a global average resolution of 7.7 Å in CRYOSPARC-2[49]. This class was classified in Relion-3 without alignment to identify stable sub-populations of the structure that would refine to higher resolution, however. Refinement of each class did not result in a reconstruction at a resolution higher than 7.7 Å. Density corresponding to the dimeric hairpin domain was subtracted from the total 100,307 particle images and the remaining density was locally refined to 5.9 Å resolution. **b** SD200i SEC trace of XPF-ERCC1-DNA complex. mAU (A_{280} nm) in blue on the Y axis. Fractions indicated in grey on the X axis. **c** Electrophoretic mobility shift assay **(**EMSA) showing XPF-ERCC1 mass shift upon binding a stem-loop DNA. Fractions A2-A12 from a SD200i SEC trace were assayed for DNA binding following mild crosslinking with BS3. Samples A4 and A10 were concentrated to 1.5 mg/ml and run on the final two lanes. The position of the DNA-bound complex and free XPF-ERCC1 are indicated. Fraction A10 was used for cryo-EM analysis.

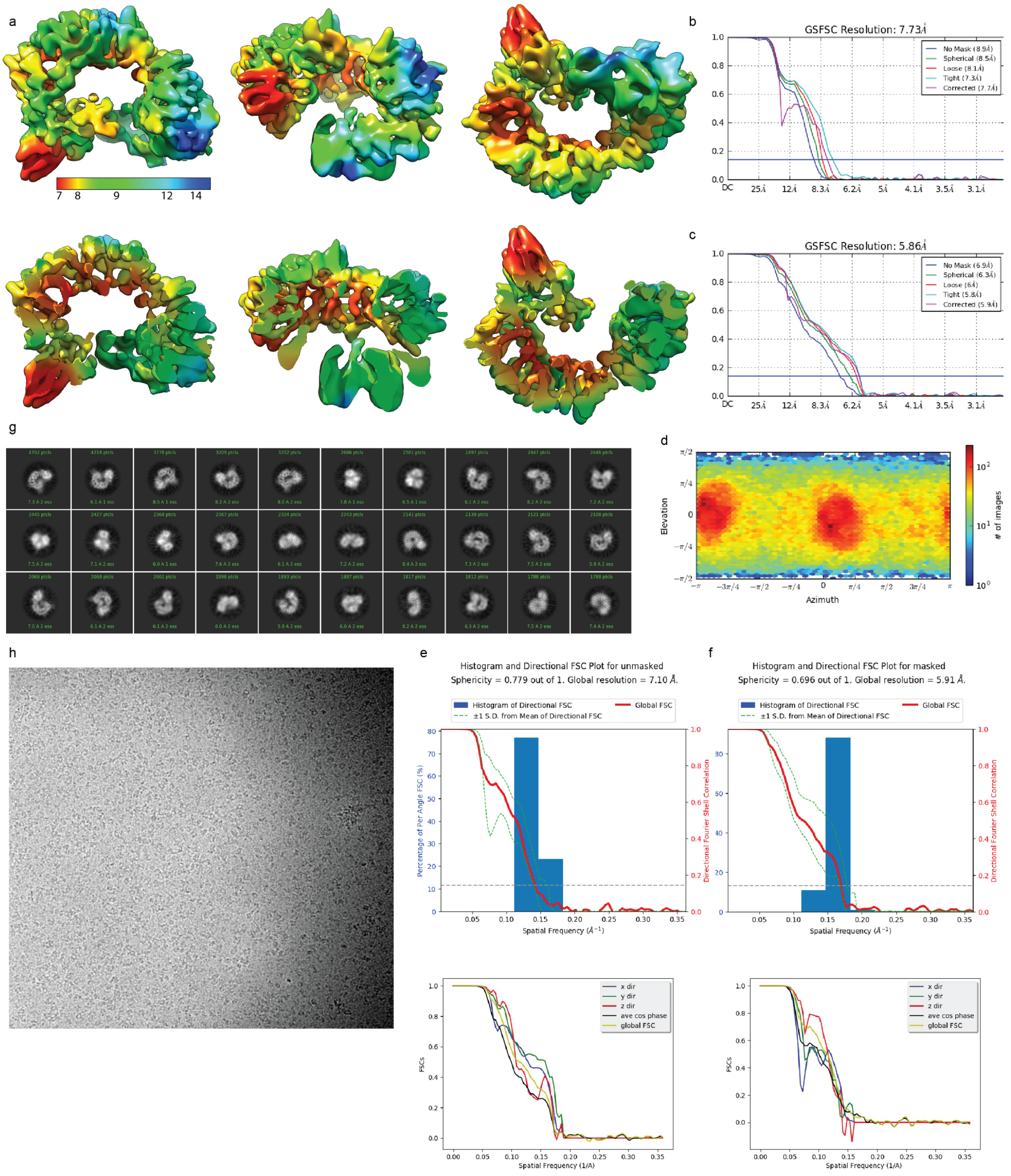

Supplementary Figure 6. XPF-ERCC1-DNA local resolution and reconstruction quality. **a** (Top) Three orthogonal views of density of the XPF-ERCC1-DNA composite map rendered based on local resolution. (Bottom) same three orthogonal views but with density cut-away to reveal inner map features. XPF RecA1, RecA2, helical and nuclease domains and ERCC1 NLD derived from local refinement omitting the 2x(HhH)$_2$ hairpins and dsDNA at 5.9 Å. XPF and ERCC1 hairpins and DNA from global refinement at 7.7 Å resolution. **b** Fourier-shell correlation (FSC) curve for global refinement at 7.7 Å resolution. **c** FSC curve for local refinement at 5.9 Å resolution. **d** Distribution of particle images contributing to global

reconstruction in CRYOSPARC-2[49] displayed in 2D. **e-f** 3D-FSC analysis for the global (unmasked) and locally (masked) refined XPF-ERCC1-DNA structures. **e** Global 3D-FSC analysis **f** Local 3D-FSC analysis **h** Motion-corrected cryo-EM micrograph of the XPF-ERCC1-DNA complex. 1.38 Å/pixel collected using a K2 detector on a Titan Krios microscope. **g** Representative selection of cryo-EM 2D class-averages of XPF-ERCC1-DNA calculated using CRYOSPARC-2[49] after final 3D classification. A mask with a diameter of 130 Å was applied. **h** Motion-corrected cryo-EM micrograph of the XPF-ERCC1-DNA complex acquired with a Titan Krios microscope at 300 keV, 1.38 Å pixel size and 3.3 μm defocus.

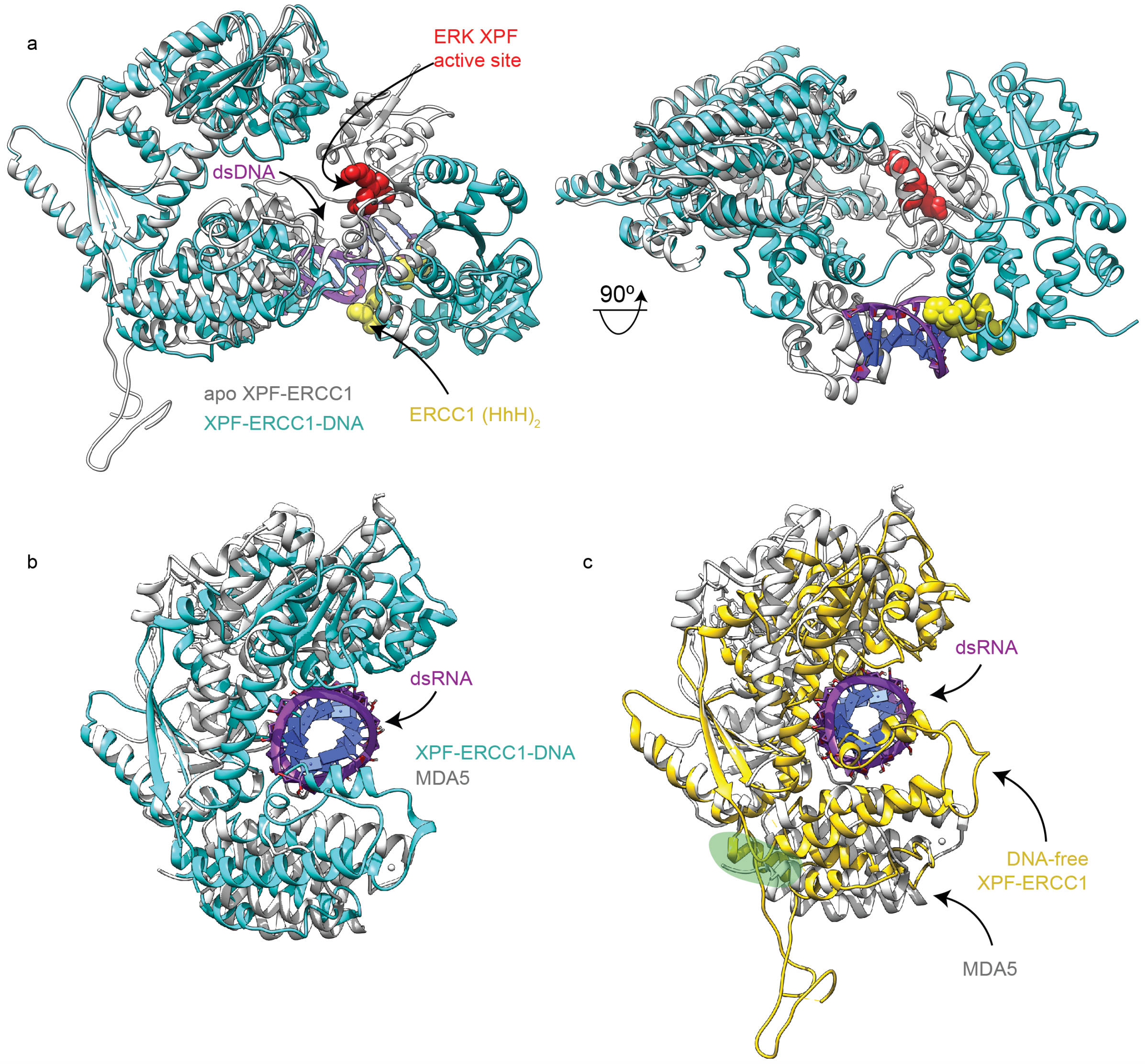

Supplementary Figure 7. Structural superpositions of XPF-ERCC1 with MDA5 helicase. **a** Structural comparison of XPF-ERCC1 in the presence and absence of stem-loop DNA. DNA-free XPF-ERCC1 in grey, overlaid with stem-loop DNA-bound XPF-ERCC1 in cyan. Labels indicate location of key features on the XPF-ERCC1-DNA model. **b** Structural superposition of the HLM of the XPF-ERCC1-DNA complex with dsRNA-bound MDA5. MDA5 (PDB code: 4GL2, residues 720-739) was structurally aligned to the XPF RecA2 domain beta sheet (residues 406-425). DNA-free XPF-ERCC1 in cyan overlaid with dsRNA-bound MDA5 in grey. dsRNA from MDA5 in purple. **c** Structural superposition of the HLM of the DNA-free XPF-ERCC1 complex with dsRNA-bound MDA5. DNA-free XPF-ERCC1 model in yellow, dsRNA-bound MDA5 in grey. dsRNA from MDA5 in purple. The α17 helix observed in DNA-free XPF-ERCC1 is shown in green. Superposition of XPF and MDA5 through their equivalent RecA2 domain sheet indicated the RecA1 domain is rotated by 25° relative to the equivalent RecA domain of dsRNA-bound MDA5 (rmsd 11.9 Å). The RecA1 ß-sheet is translated by

~15Å from the MDA5 equivalent RecA1 ß-sheet. The RecA2 domain α17 helix orientation is perpendicular to the direction of the equivalent helix (residues 742-746) of MDA5. The positioning of α17 locks the XPF helical domain in place, rotated by 45° relative to the equivalent domain in MDA5, in a conformation that is not conducive to dsDNA-binding.

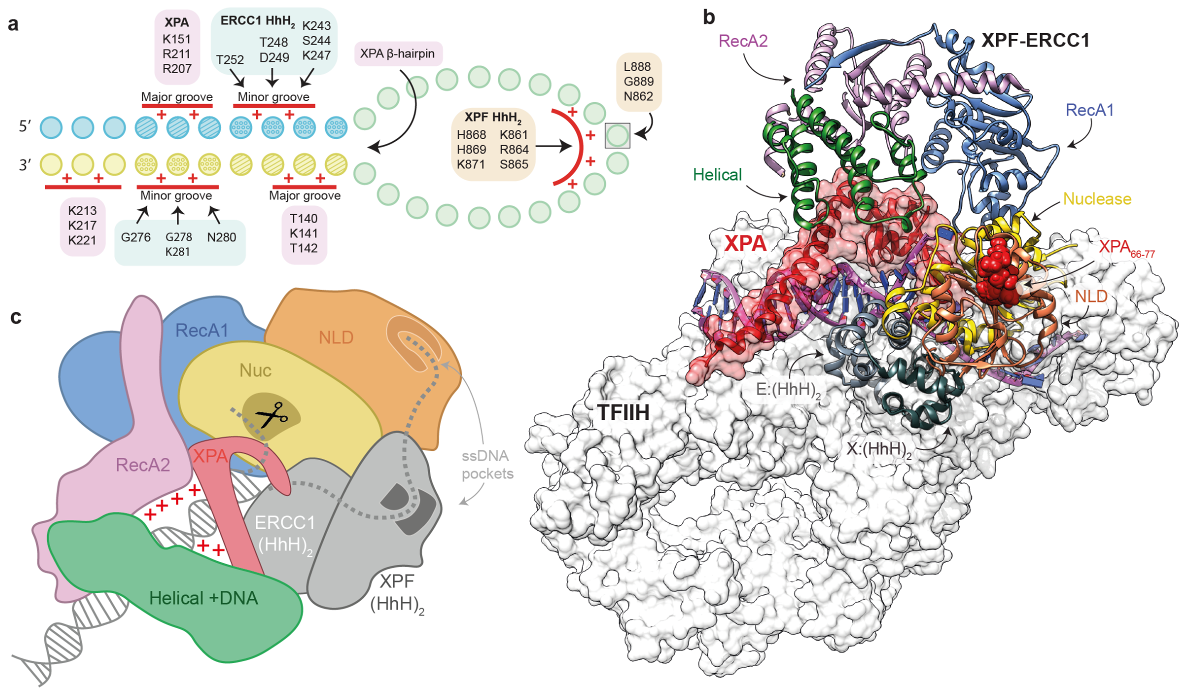

Supplementary Figure 8. Model for a complete 5'-NER incision complex. a Schematic of XPF-ERCC1 and XPA interactions with a stem-loop DNA substrate, obtained by combining information from this study and from PDB code 6RO4. XPA interaction elements are displayed in pink, ERCC1 interactions displayed in pale blue and XPF interactions in orange. There is a notable complementarity of XPA and XPF-ERCC1 binding to the splayed arm DNA and no mutually overlapping contacts consistent with a synergistic interaction. **b** Model for XPF-ERCC1 binding to ds/ssDNA-bound TFIIH-XPA. Stem-loop DNA-bound XPF-ERCC1 model was docked onto the TFIIH-XPA-splayed arm DNA structure (PDB: 6RO4) through the exposed dsDNA minor groove at the ss/dsDNA boundary. The XPF-ERCC1 model is coloured according to domain as in Fig. 1a, DNA (purple), XPA (red) displayed with transparent surface rendering. TFIIH is displayed in grey with opaque surface rendering. **c** Cartoon schematic of hypothetical, fully activated XPF-ERCC1 in which the major groove upstream of the ERCC1 $(HhH)_2$ domain is engaged by the concave surface of the XPF HLM which feeds the DNA substrate into the active site. XPA is shown separating the strands of a dsDNA duplex.

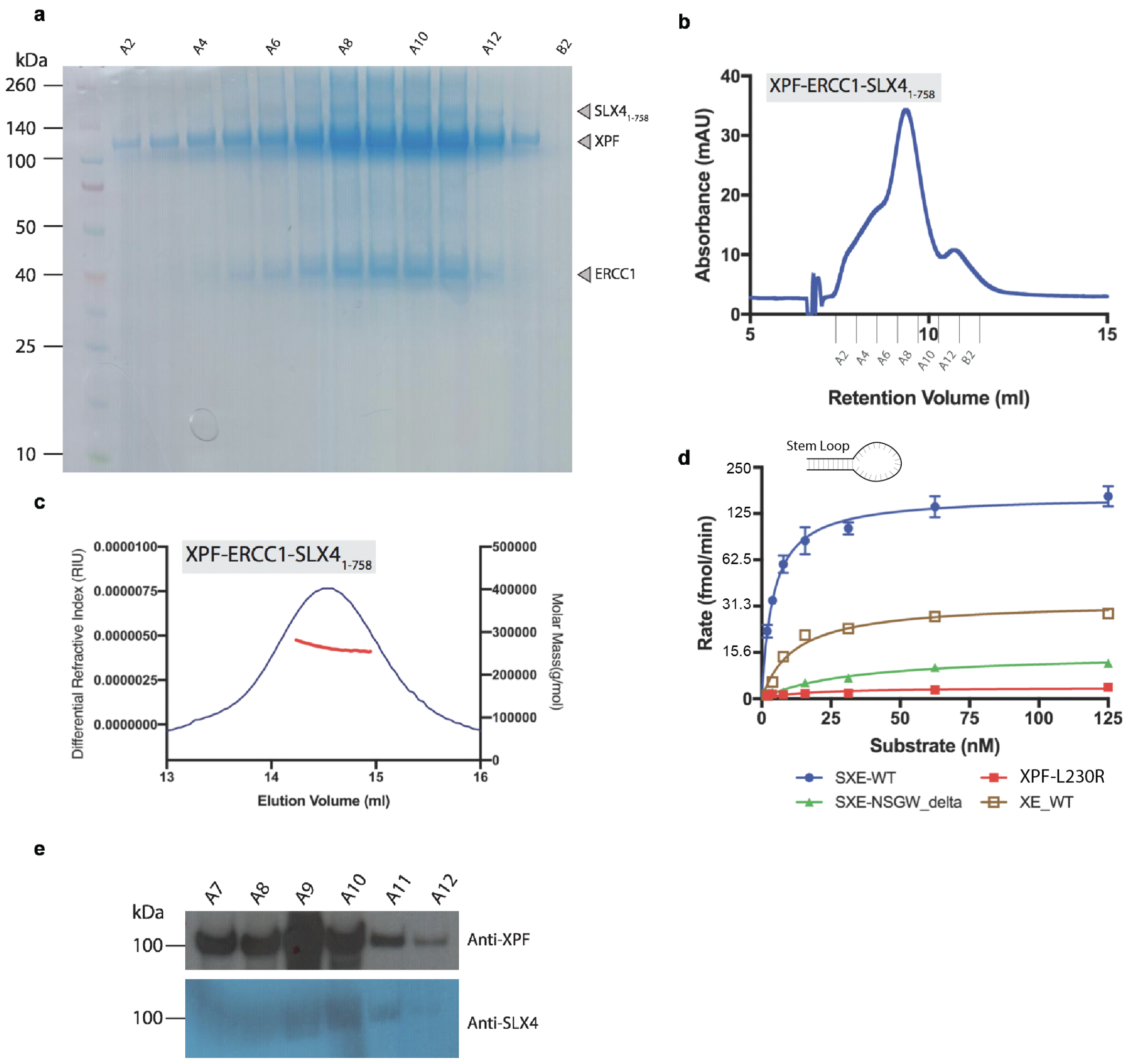

Supplementary Figure 9. Characterisation of XPF-ERCC1-SLX4^{NTD} complexes a SDS-PAGE gel of the XPF-ERCC1-SLX4^{NTD} complex. **b** Superose-6 increase SEC column trace for purified XPF-ERCC1-SLX4^{NTD} complex. **c** SEC-MALLS for the XPF-ERCC1-SLX4^{NTD} complex. A molecular weight estimate of 240 kDa from SEC-MALLS indicates the complex has a 1:1:1 stoichiometry. **d** Michaelis-Menten plot of rate vs substrate concentration for XPF-ERCC1 wild-type (XE_WT), XPF-ERCC1-SLX4^{NTD} (SXE_WT), XPF$^{NSGW\Delta}$-ERCC1-SLX4^{NTD} (SXE_NSGW_delta). The Superose-6 increase column fractions at the same retention volume to those that contained XPF-ERCC1-SLX4^{NTD} wild-type complex were assayed for the XPF$^{NSGW\Delta}$-ERCC1-SLX4^{NTD} mutant. These fractions contained a substantially reduced incision activity than XE_WT alone. Error bars indicate standard deviation (n=3). Kinetic values available in Table 3. **e** Western blot showing fractions for XPF-ERCC1-SLX4^{NTD} contain both XPF and SLX4.

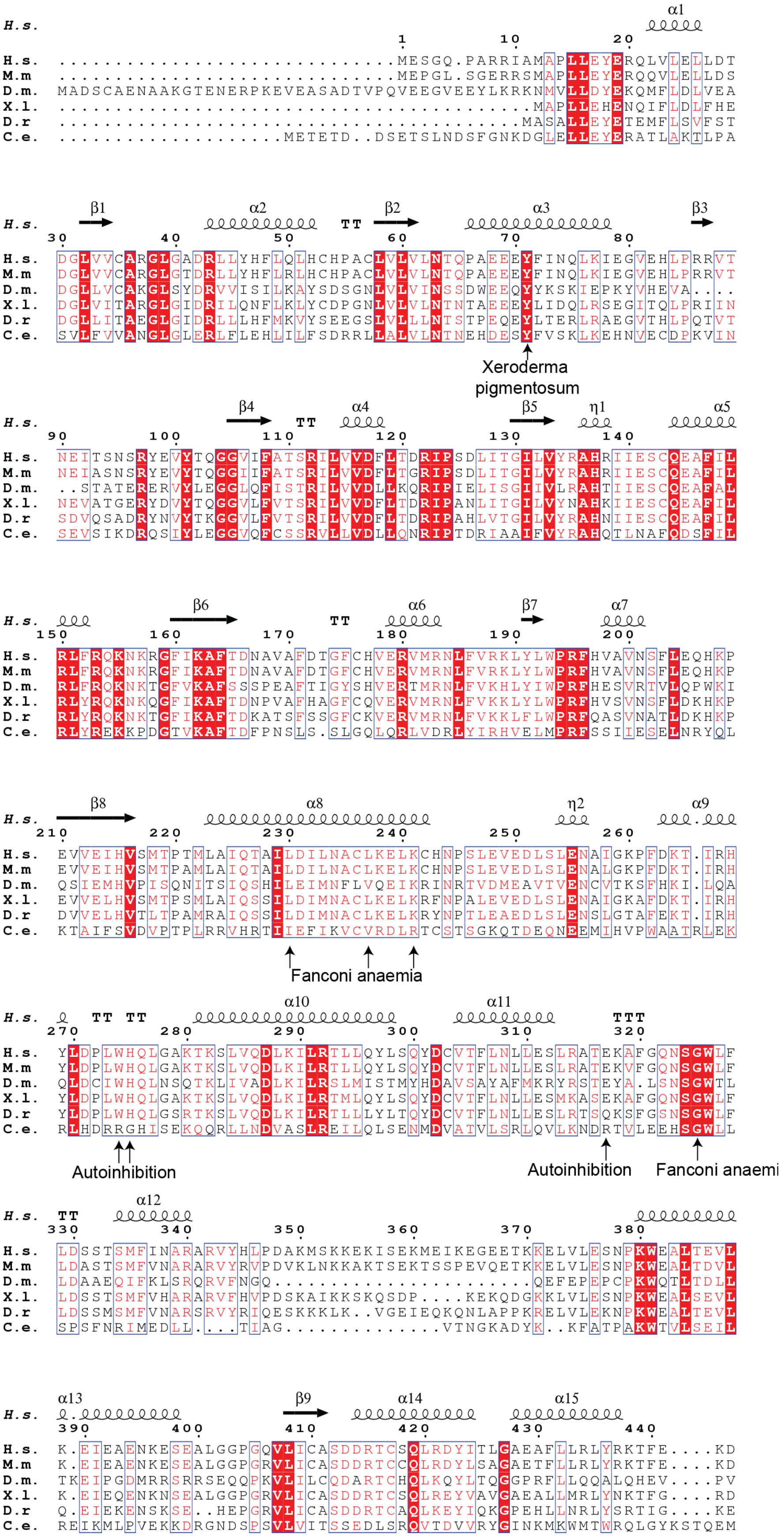

H.s. α1
1 10 20
H.s. ....MESGQ.PARRIAMAPLLEYERQLVLELLDT
M.m ....MEPGL.SGERRSMAPLLEYERQQVLELLDS
D.m. MADSCAENAAKGTENERPKEVEASADTVPQVEEGVEEYLKRKNMVLLDYEKQMFLDLVEA
X.l. ....MAPLLEHENQIFLDLFHE
D.r ....MASALLEYETEMFLSVFST
C.e. ....METETD..DSETSLNDSFGNKDGLELLEYERATLAKTLPA
H.s. β1 α2 TT β2 α3 β3
30 40 50 60 70 80
H.s. DGLVVCARGLGADRLLYHFLQLHCHPACLVLVLNTQPAEEEYFINQLKIEGVEHLPRRVT
M.m DGLVVCARGLGTDRLLYHFLRLHCHPACLVLVLNTQPAEEEYFINQLKIEGVEHLPRRVT
D.m. DGLLVCAKGLSYDRVVISILKAYSDSGNLVLVINSSDWEEQYYKSKIEPKYVHEVA...
X.l. DGLVITARGLGIDRILQNFLKLYCDPGNLVLVLNTNTAEEEYLIDQLRSEGITQLPRIIN
D.r DGLLITAEGLGIDRLLLHFMKVYSEEGSLVLLLNTSTPEQEYLTERLRAEGVTHLPQTVT
C.e. SVLFVVANGLGLERLFLEHLILFSDRRLLALVLNTNEHDESYFVSKLKEHNVECDPKVIN
Xeroderma pigmentosum
H.s. β4 TT α4 β5 η1 α5
90 100 110 120 130 140
H.s. NEITSNSRYEVYTQGGVIFATSRILVVDFLTDRIPSDLITGILVYRAHRIIESCQEAFIL
M.m NEIASNSRYEVYTQGGIIFATSRILVVDFLTGRIPSDLITGILVYRAHRIIESCQEAFIL
D.m. ..STATERERVYLEGGLQFISTRILVVDLLKQRIPIELISGIIVLRAHTIIESCQEAFAL
X.l. NEVATGERYDVYTQGGVLFVTSRILVVDFLTDRIPANLITGILVYNAHKIIESCQEAFIL
D.r SDVQSADRYNVYTKGGVLFVTSRILVVDFLTDRIPAHLVTGILVYRAHNIIESCQEAFIL
C.e. SEVSIKDRQSIYLEGGVQFCSSRVLVVDLLQNRIPTDRIAAIFVYRAHQTLNAFQDSFIL
H.s. β6 TT α6 β7 α7
150 160 170 180 190 200
H.s. RLFRQKNKRGFIKAFTDNAVAFDTGFCHVERVMRNLFVRKLYLWPRFHVAVNSFLEQHKP
M.m RLFRQKNKRGFIKAFTDNAVAFDTGFCHVERVMRNLFVRKLYLWPRFHVAVNSFLEQHKP
D.m. RLFRQKNKTGFVKAFSSSPEAFTIGYSHVERTMRNLFVKHLYIWPRFHESVRTVLQPWKI
X.l. RLYRQKNKQGFIKAFTDNPVAFHAGFCQVERVMRNLFVKKLYLWPRFHVSVNSFLDKHKP
D.r RLYRQKNKTGFIKAFTDKATSFSSGFCKVERVMRNLFVKKLFLWPRFQASVNATLDKHKP
C.e. RLYREKKPDGTVKAFTDFPNSLS.SLGQLQRLVDRLYIRHVELMPRFSSIIESELNRYQL
H.s. β8 α8 η2 α9
210 220 230 240 250 260
H.s. EVVEIHVSMTPTMLAIQTAILDILNACLKELKCHNPSLEVEDLSLENAIGKPFDKT.IRH
M.m EVVEIHVSMTPAMLAIQTAILDILNACLKELKCHNPSLEVEDLSLENALGKPFDKT.IRH
D.m. QSIEMHVPISQNITSIQSHILEIMNFLVQEIKRINRTVDMEAVTVENCVTKSFHKI.LQA
X.l. EVVELHVSMTPSMLAIQSSILDIMNACLKELKRFNPALEVEDLSLENAIGKAFDKT.IRH
D.r DVVELHVTLTPAMRAIQSSILDIMNACLKELKRYNPTLEAEDLSLENSLGTAFEKT.IRH
C.e. KTAIFSVDVPTPLRRVHRTIIEFIKVCVRDLRTCSTSGKQTDEQNEEMIHVPWAATRLEK
Fanconi anaemia
H.s. TT TT α10 α11 TTT
270 280 290 300 310 320
H.s. YLDPLWHQLGAKTKSLVQDLKILRTLLQYLSQYDCVTFLNLLESLRATEKAFGQNSGWLF
M.m YLDPLWHQLGAKTKSLVQDLKILRTLLQYLSQYDCVTFLNLLESLRATEKVFGQNSGWLF
D.m. QLDCIWHQLNSQTKLIVADLKILRSLMISTMYHDAVSAYAFMKRYRSTEYA.LSNSGWTL
X.l. YLDPLWHQLGAKTKSLVQDLKILRTMLQYLSQYDCVTFLNLLESMKASEKAFGQNSGWLF
D.r YLDPLWHQLGSRTKSLVQDLKILRTLLLYLTQYDCVTFLNLLESLRTSQKSFGSNSGWLF
C.e. RLHDRRGHISEKQQRLLNDVASLREILQLSENMDVATVLSRLQVLKNDRTVLEEHSGWLL
Autoinhibition
Autoinhibition
Fanconi anaemi
H.s. TT α12
330 340 350 360 370 380
H.s. LDSSTSMFINARARVYHLPDAKMSKKEKISEKMEIKEGEETKKELVLESNPKWEALTEVL
M.m LDASTSMFVNARARVYRVPDVKLNKKAKTSEKTSSPEVQETKKELVLESNPKWEALTDVL
D.m. LDAAEQIFKLSRQRVFNGQ.......................QEFEPEPCPKWQTLTDLL
X.l. LDSSTSMFVHARARVFHVPDSKAIKKKSQSDP....KEKQDGKKLVLESNPKWEALSEVL
D.r LDSSMSMFVNARSRVYRIQESKKKLK..VGEIEQKQNLAPPKRELVLEKNPKWEALTEVL
C.e. SPSFNRIMEDLL....TIAG..............VTNGKADYK..KFATPAKWTVLSEIL
H.s. α13 β9 α14 α15
390 400 410 420 430 440
H.s. K.EIEAENKESEALGGPGQVLICASDDRTCSQLRDYITLGAEAFLLRLYRKTFE....KD
M.m K.EIEAENKESEALGGPGRVLICASDDRTCCQLRDYLSAGAETFLLRLYRKTFE....KD
D.m. TKEIPGDMRRSRRSEQQPKVLILCQDARTCHQLKQYLTQGGPRFLLQQALQHEV....PV
X.l. K.EIEQENKNSEALGGPGRVLICASDDRTCSQLREYVAVGAEALLMRLYNKTFG....RD
D.r Q.EIEKENSKSE..HEPGRVLICASDDRTCAQLKEYIQKGPEHLLNRLYSRTIG....KE
C.e. REIKMLPVEKKDRGNDSPSVLVITSSEDLSRQVTDVVRYGINKMKWMTWRQLGYKSTQEM

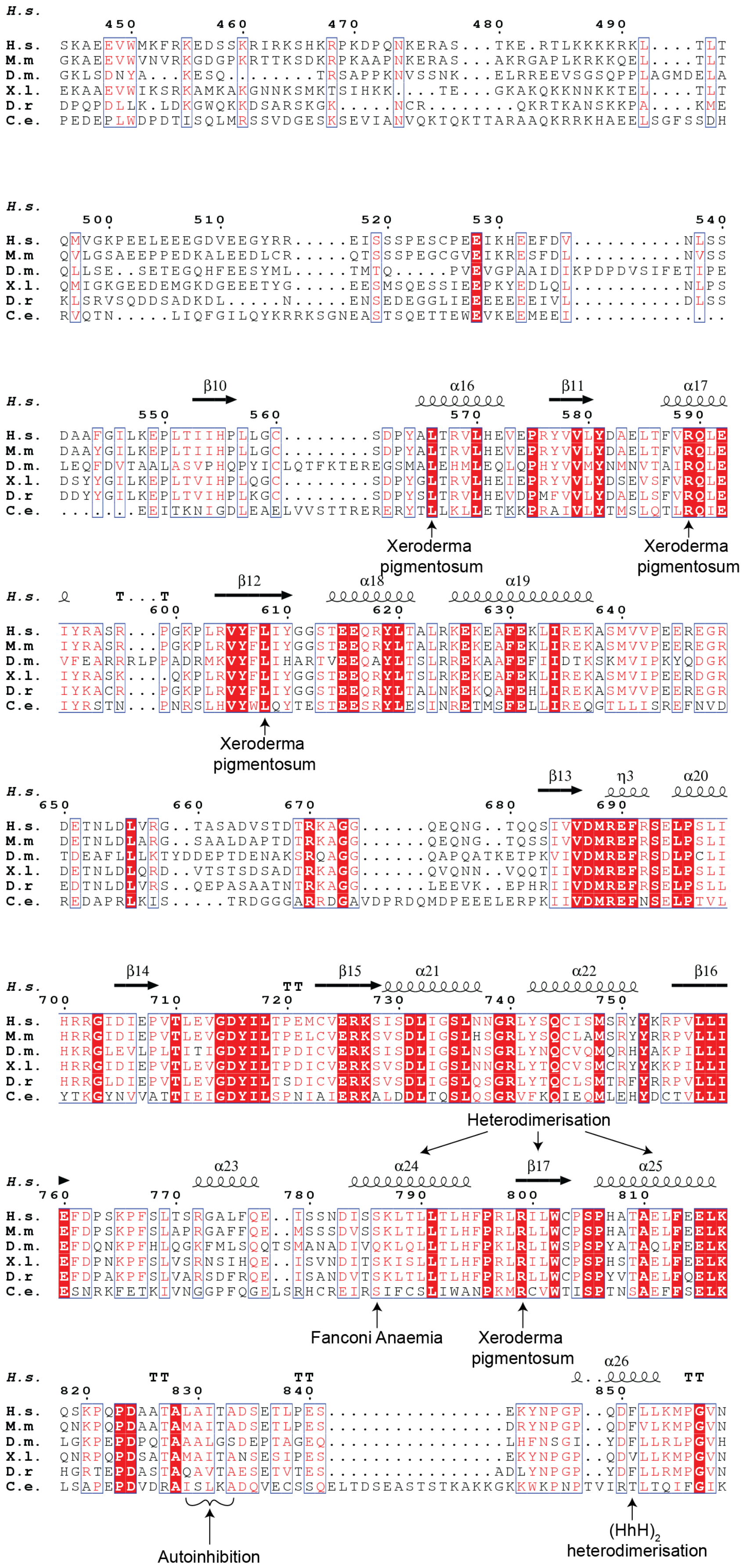
Xeroderma pigmentosum
Xeroderma pigmentosum
Xeroderma pigmentosum
Heterodimerisation
Fanconi Anaemia
Xeroderma pigmentosum
Autoinhibition
(HhH)2 heterodimerisation

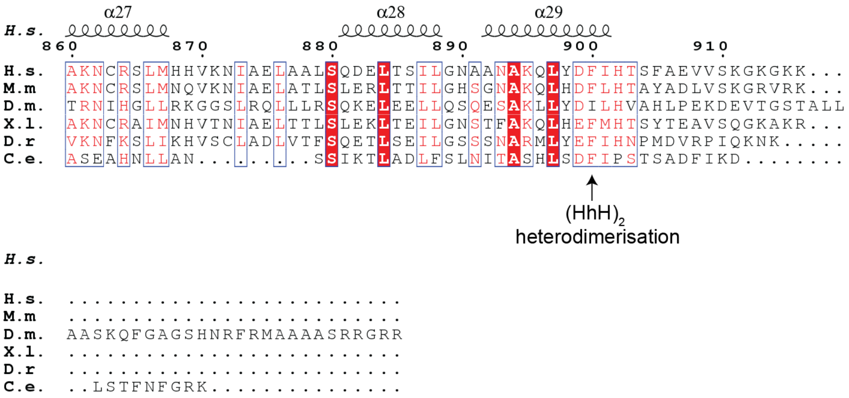

Supplementary Figure 10. Multiple sequence alignments for XPF. *H. sapiens (H.s.), Mus musculus (M.m.), D. melanogaster (D.m.), C. elegans (C.e.), D. rerio (D. r.)* and *X. laevis (X.l.)* XPF sequences aligned with secondary structural elements above the alignment shown and annotated as used throughout the text. Residues are coloured by residue type and invariant residues indicated or highlighted by conservation.

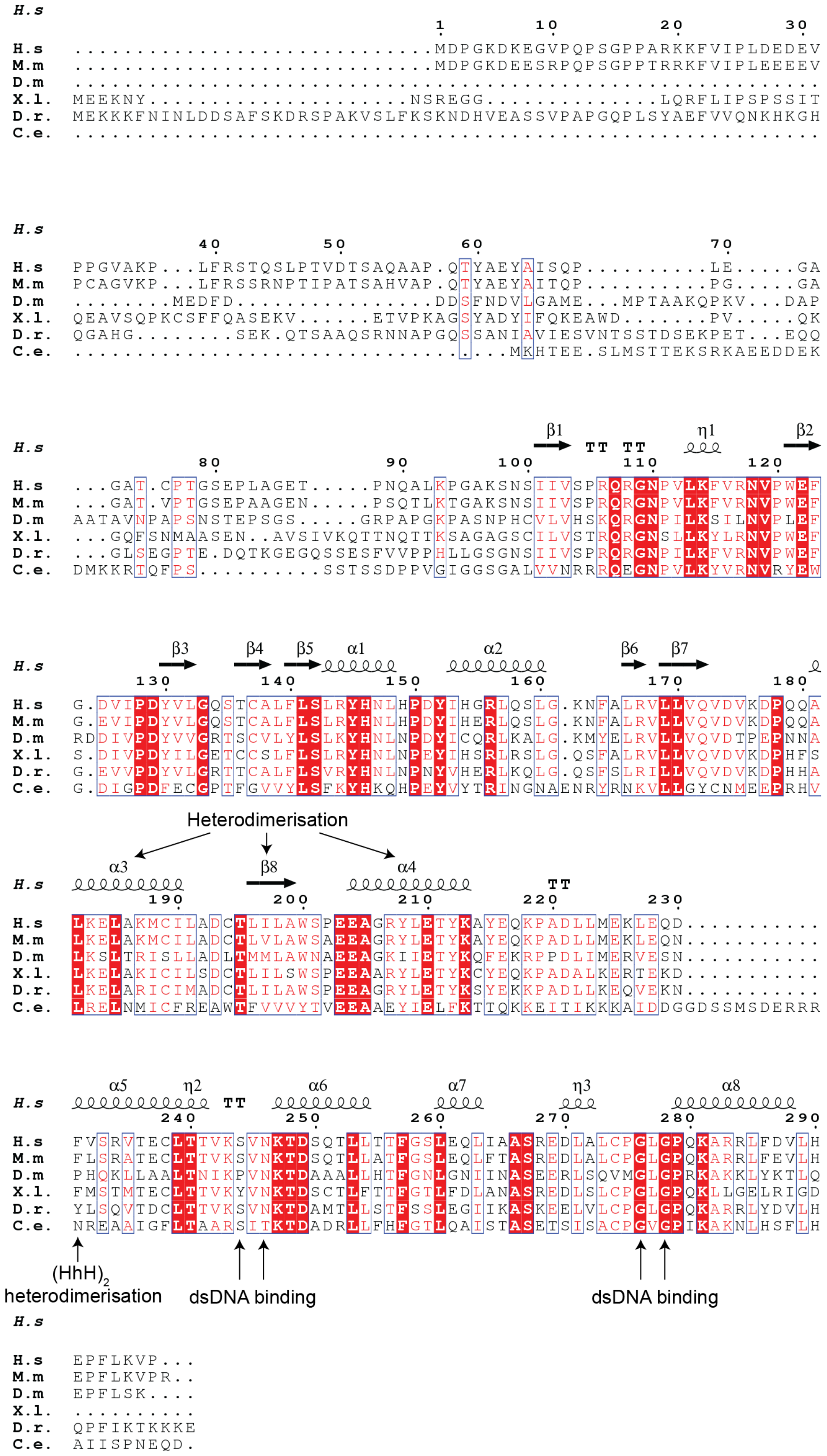

Supplementary Figure 11. Multiple sequence alignments for ERCC1. *H. sapiens (H.s.), Mus musculus (M.m.), D. melanogaster (D.m.), C. elegans (C.e.), D. rerio (D. r.)* and *X. laevis (X.l.)* ERCC1 sequences aligned with secondary structural elements above the alignment shown and annotated as used throughout the text. Residues are coloured by residue type and invariant residues indicated or highlighted by conservation.

Supplementary Tables

Supplementary Table 1.

XPF Residues	Wild Type Sequence	Modelled
156-159	NKRG	Poly-alanine
170-172	AFD	Poly-alanine
174-177	GFCH	Poly-alanine
250-254	EDLSL	Poly-alanine
322-323	QN	Poly-alanine
345-374	HLPDAKMSKKEKISEKMEIKEGEETKKELV	Poly-alanine
396-403	KESEALGG	Poly-alanine
440-441	FE	Poly-alanine
582	D	Poly-alanine
640-641	MV	Poly-alanine
680	T	Poly-alanine
771-780	SRGALFQEIS	Poly-alanine
824-845	DAATALAITADSETLPESEKYN	Poly-alanine
441-550	EKDSKAEEVWMKFRKEDSSKRIRKSHKRPKD PQNKERASTKERTLKKKKRKLTLTQMGKPEE LEEEGDVEEGYRREISSSPESCPEEIKHEEFD VNLSSDAAFGILKEP	Not modelled
642-679	VPEEREGRDETNLDLVRGTASADVSTDTRKA GGQEQNG	Not modelled
907-916	EVVSKGKGKK	Not modelled

ERCC1 Residues	Wild Type Sequence	Modelled
223-229	LMEKLEQ	Poly-alanine
293-297	FLKVP	Poly-alanine
1-99	MDPGKDKEGVPQPSGPPARKKFVIPLDEDEV PPGVAKPLFRSTQSLPTVDTSAQAAPQTYAE YAISQPLEGAGATCPTGSEPLAGETPNQALKP GAKSN	Not modelled, Likely to be clipped from protein

Omitted regions from the final XPF-ERCC1 model. Regions in XPF modelled as poly-alanine or not included in the model. Regions in ERCC1 modelled as poly-alanine or not included in the model**.**

Supplementary Table 2.

Id	Score	Prot1	APos1	PepSeq1	LPos1	Prot2	APos2	PepSeq2	LPos2	Distance
16	339.33	ERCC1	243	VTECLTTVKSVNK	9	XPF	289	SLVQDLKILR	7	22.1912819
116	309.73	XPF	911	QLYDFIHTSFAEVVSKGK	16	ERCC1	218	AYEQKPADLLMEK	5	41.3972278
113	289.62	XPF	632	EKEAFEKLIR	7	ERCC1	114	GNPVLKFVR	6	65.3133353
152	286.7	XPF	364	MEIKEGEETKK	4	XPF	356	KEKISEK	3	14.7854835
63	275.28	XPF	350	VYHLPDAKMSK	8	XPF	356	KEKISEK	3	29.0647298
17	270.67	ERCC1	243	VTECLTTVKSVNK	9	XPF	282	TKSLVQDLKILR	2	26.6985929
6	259.4	XPF	350	VYHLPDAKMSK	8	XPF	360	EKISEKMEIK	6	27.6324186
20	255.31	ERCC1	226	AYEQKPADLLMEKLEQDFVSR	13	XPF	861	MPGVNAKNCR	7	39.8053336
162	255.11	XPF	445	TFEKDSKAEEVWMK	7	XPF	364	MEIKEGEETKK	4	25.3386126
143	236.42	ERCC1	176	VLLVQVDVKDPQQALK	9	XPF	627	EKEAFEK	2	58.4867486
65	231.26	XPF	365	MEIKEGEETKK	4	XPF	350	VYHLPDAKMSK	8	20.2729493
151	228.65	XPF	365	MEIKEGEETKK	4	XPF	442	TFEKDSK	4	25.3386126
130	227.77	XPF	442	KTFEKDSKAEEVWMK	5	XPF	360	ISEKMEIK	4	32.2970996
71	224.61	XPF	280	HYLDPLWHQLGAKTK	13	ERCC1	114	GNPVLKFVR	6	47.7163459
164	213.45	ERCC1	114	GNPVLKFVR	6	XPF	442	KTFEK	1	80.4789278
138	213.45	ERCC1	162	LQSLGKNFALR	6	XPF	861	MPGVNAKNCR	7	53.9522986
13	212.28	XPF	637	EKASMVVPEER	2	XPF	189	KLYLWPR	1	12.2904222
125	202.3	ERCC1	281	EDLALCPGLGPQKAR	13	ERCC1	243	VTECLTTVKSVNK	9	22.6149431
167	201.49	ERCC1	114	GNPVLKFVR	6	XPF	627	EKEAFEK	2	65.3133353
158	199.68	XPF	637	EKASMVVPEER	2	XPF	627	EKEAFEK	2	16.1306385
70	197.59	ERCC1	176	VLLVQVDVKDPQQALK	9	ERCC1	114	GNPVLKFVR	6	21.1747562
51	192.26	XPF	637	EKASMVVPEER	2	ERCC1	114	GNPVLKFVR	6	60.9392554
146	191.24	ERCC1	281	EDLALCPGLGPQKAR	13	XPF	289	SLVQDLKILR	7	30.0542173
114	190.73	XPF	260	CHNPSLEVEDLSLENAIGKPFDKTIR	23	ERCC1	243	VTECLTTVKSVNK	9	36.893582
18	188.3	ERCC1	218	AYEQKPADLLMEK	5	ERCC1	114	GNPVLKFVR	6	24.8052164
144	187.73	XPF	854	YNPGPQDFLLKMPGVNAK	11	ERCC1	243	VTECLTTVKSVNK	9	22.7149549
75	186.24	XPF	753	YYKRPVLLIEFDPSKPFSLTSR	3	ERCC1	162	LQSLGKNFALR	6	28.6550138
172	185.87	XPF	318	ATEKAFGQNSGWLFLDSSTSMFINAR	4	XPF	364	MEIKEGEETKK	4	25.3797966

74	183.67	XPF	765	RPVLLIEFDPSKPFSLTSR	12	XPF	282	TKSLVQDLK	2	40.2171883
120	183.63	XPF	787	GALFQEISSNDISSKLTLLTLHFPR	15	ERCC1	218	AYEQKPADLLMEK	5	19.6340958
46	178.42	XPF	396	EIEAENKESEALGGPGQVLICASDDR	7	XPF	601	ASRPGKPLR	6	27.2329106
81	177.14	XPF	787	GALFQEISSNDISSKLTLLTLHFPR	15	XPF	861	MPGVNAKNCR	7	43.0332262
115	175.02	XPF	753	YYKRPVLLIEFDPSKPFSLTSR	3	XPF	861	MPGVNAKNCR	7	31.3034027
44	174.86	ERCC1	218	AYEQKPADLLMEK	5	XPF	861	MPGVNAKNCR	7	39.8053336
80	169.4	XPF	765	YYKRPVLLIEFDPSKPFSLTSR	15	ERCC1	176	VLLVQVDVKDPQQALK	9	24.0908409
173	158.12	XPF	318	ATEKAFGQNSGWLFLDSSTSMFINAR	4	XPF	356	EKISEK	2	35.780336
39	156.48	XPF	365	MEIKEGEETK	4	XPF	371	KELVLESNPK	1	12.6424059
76	156.23	XPF	260	CHNPSLEVEDLSLENAIGKPFDKTIR	19	XPF	289	SLVQDLKILR	7	14.8785705
98	152.72	XPF	350	VYHLPDAKMSK	8	XPF	354	KEKISEK	1	29.0647298
53	151.83	ERCC1	176	VLLVQVDVKDPQQALK	9	XPF	861	MPGVNAKNCR	7	44.3778939
92	147.46	XPF	365	MEIKEGEETKK	4	XPF	353	MSKKEK	3	13.1828978
77	136.48	XPF	260	CHNPSLEVEDLSLENAIGKPFDKTIR	23	XPF	289	SLVQDLKILR	7	14.8785705
43	132.32	XPF	353	VYHLPDAKMSKKEK	11	XPF	371	KELVLESNPK	1	10.0961676
78	105.14	XPF	280	HYLDPLWHQLGAKTK	13	XPF	289	SLVQDLKILR	7	17.9187822
73	93.6	ERCC1	114	GNPVLKFVR	6	XPF	189	KLYLWPR	1	65.8048229
79	62.72	XPF	765	YYKRPVLLIEFDPSKPFSLTSR	15	ERCC1	162	LQSLGKNFALR	6	28.6550138

Crosslinking mass spectrometry. All verified XL-MS crosslinks displayed. Id – unique identifier, score – normalised prevalence value representing the frequency of crosslink appearance in MS2, Prot1/2 – protein from which the respective crosslink originates, APos1/2 – position in sequence for respective crosslink, Pseq1/2 – protein sequence identified in MS1, LPos1/2 – link position identifying which residue in Pseq the crosslink maps to.

Supplementary Table 3.

Protein	Construct	Forward Primer	Reverse Primer
XPF	L230R	GACTGCTATACGGGACATTTTAAATG	TGTATAGCCAAGCATGGTAG
XPF	L236R	TTTAAATGCACGCCTAAAGGAACTAAAATG	ATGTCCAGTATAGCAGTC
XPF	E239K	ATGTCTAAAGAAACTAAAATGCCATAAC	GCATTTAAAATGTCCAGTATAG
XPF	S786F	TGACATTAGTTTCAAACTCACTCTTC	TTGCTGGAGATCTCCTGAAAC
XPF	323-326 Δ	CTGTTTCTTGACTCCAGC	CTGACCAAAAGCTTTTTC
XPF	S312A	TCTTCTGGAAGCTCTGAGAGCAA	TTAAGAAATGTGACACAATCATACTG
XPF	W274A, H275A	GGATCCTTTGGCGGCCCAGCTTGGAG	AGATAATGGCGGATTGTC
XPF	R112A	TGCGACAAGTGCAATACTTGTGG	AATATAACACCACCTTGTG
XPF	829-833 Δ	GCCATTACAGCAGATTCTGAAACCC	CGCCGCATCAGGCTGTGG
XPF	R589W	AACCTTTGTTTGGCAGCTTGAAA	AGCTCTGCGTCATAAAGAAC
XPF	Q300A	GTATCTCTCTGCTTATGATTGTGTCACATTTCTTAATC	TGCAGCAAAGTTCGTAATATC
XPF	L608P	GGTTTACTTTCCTATATACGGAGG	CTCAGAGGTTTCCCAGGC
XPF	T567A	CTATGCTCTGGCTAGGGTACTACATGAAGTG	GGGTCGCTGCAACCCAGA
XPF	Y71A	CGAGGAGGAGGCTTTTATCAATCAGCTGAAGATAGAAGGAGTTGAAC	GCCGGCTGCGTGTTGAGC
XPF	R799W	CCCCAGACTATGGATTCTCTGGT	AAGTGAAGTGTAAGAAGAGTGAG
ERCC1	L253A	CAGTCAGACCGCCCTGACCACATTTG	TCCGTTTTGTTGACTGAC
ERCC1	T248A	AGTCAACAAAGCTGACAGTCAGACC	GACTTCACGGTGGTCAGA
ERCC1	T252A	GGACAGTCAGGCTCTCCTGACCA	GTTTTGTTGACTGACTTCACGG
ERCC1	NheI for Strep tag	ATCCAGCTAGCGAGAAAACCTGTACTTCCAGTCATGGAGCC	ATTCAGCTAGCCCCTTCTCGAACTGAGGGTGAGACCAAGAAC

Primer sequences used in this study
