# Supplementary material for: Cryo-EM structures of the XPF-ERCC1 endonuclease reveal how DNA-junction engagement disrupts an auto-inhibited conformation
Source: Nat Commun. 2020 Feb 28;11:1120. doi: 10.1038/s41467-020-14856-2 (PMC7048804; doi:10.1038/s41467-020-14856-2)
Supplement: Supplementary file 2 — Description of Additional Supplementary Files [file 41467_2020_14856_MOESM2_ESM.pdf]

Description of Additional Supplementary Files

File Name: Supplementary Movie 1
Description: **DNA-free XPF-ERCC1 local resolution.** Locally filtered DNA-free composite map obtained by global and local refinement. The colour key indicates the local resolution ranging from 3.4 – 6 Å resolution. The XPF-ERCC1 complex is first rotated in two planes and then the density is cut away to reveal the local resolution within the core of the molecule.

File Name: Supplementary Movie 2
Description: **DNA-free XPF-ERCC1 map quality and domain organisation.** Movie displays the locally filtered DNA-free composite map from global and local refinement. XPF density is displayed in purple and ERCC1 displayed in light blue. Different views of the complex are displayed to indicate the map quality and evidence of sidechains and secondary structure. The ERCC1 density is first represented as mesh, followed by the XPF density to highlight the intertwining of XPF and ERCC1 subunits. Next the map corresponding to each domain is coloured according to Figure 1a. The map then focusses on the XPF nuclease – ERCC1 NLD dimer, followed by the RecA1 – RecA2 module and finally the XPF helical – dimeric 2x(HhH)2 domain functional unit. At each stage the map is meshed out to focus the viewers eye on the domains being displayed. The map surface is then switched into a transparent mode and the ribbon model fitted to the density, colour coded by domain. The movie then cycles through the functional units as previously done for the map alone highlighting the quality of the model building.

File Name: Supplementary Movie 3
Description: **DNA-free XPF-ERCC1 autoinhibition interfaces.** Ribbon model of XPF-ERCC1 coloured by domain according to Figure 1a. Movie first highlights the autoinhibition interface formed between the XPF helical and nuclease domains centered around H275XPF, W274XPF and S730XPF. These residues are displayed in stick rendering and coloured according to heteroatom. These residues are subsequently highlighted in orange. The moive then moves to the second autoinhibition interface between the XPF helical and ERCC1 (HhH)2 domain. Residues N308XPF, S312 XPF, A315 XPF, P275 ERCC1, T252 ERCC1, T255 ERCC1 and T256 ERCC1 are highlighted in orange. The dsDNA binding motifs on ERCC1 are then highlighted in red with residues displayed as sticks. Finally, an overview of the complex is provided with transparent surface rendering to indicate the crowded molecular environment surrounding the two autoinhibition interfaces.

File Name: Supplementary Movie 4
Description: **DNA-bound XPF-ERCC1 local resolution.** DNA-bound composite map from global and local refinement coloured by local resolution, ranging from 7 – 14 Å resolution according to the key. Density is cut away in two orthogonal planes to reveal the local resolution within the core of the molecule.

File Name: Supplementary Movie 5
Description: **DNA-bound XPF-ERCC1 structural overview.** Ribbon model of DNA-bound XPF-ERCC1 coloured by domain according to Figure 1a fit into map density. The model is rotated to reveal the quality of fit throughout the molecule.

File Name: Supplementary Movie 6
Description: **Eigenvector 1 for 3D variability analysis DNA-free XPF-ERCC1.** This movie shows eigenvector 1 of the 3D covariance for the reconstruction. The eigenvectors were filtered to 6 Å resolution during optimisation to prevent noise from dominating the determination of the eigenvectors.

File Name: Supplementary Movie 7
Description: **Eigenvector 2 for 3D variability analysis DNA-free XPF-ERCC1.** This movie shows eigenvector 2 of the 3D covariance for the reconstruction. The eigenvectors were filtered to 6 Å resolution during optimisation to prevent noise from dominating the determination of the eigenvectors.

File Name: Supplementary Movie 8
Description: **Eigenvector 3 for 3D variability analysis DNA-free XPF-ERCC1.** This movie shows eigenvector 3 of the 3D covariance for the reconstruction. The eigenvectors were filtered to 6 Å resolution during optimisation to prevent noise from dominating the determination of the eigenvectors.

File Name: Supplementary Movie 9
Description: **View 1 showing domain rearrangements following substrate engagement.** View 1 morphing between the DNA-free and DNA-bound conformations of XPF-ERCC1. This highlights the substantive movement of the dimeric 2x(HhH)2 domain and the rotation by 15[illegible] of the XPF helical domain away from the XPF nuclease domain. The main conformational changes occur at the autoinhibition interfaces.

File Name: Supplementary Movie 10
Description: **View 2 showing domain rearrangements following substrate engagement.** Orthogonal view to movie 9, morphing between the DNA-free and DNA-bound conformations of XPF-ERCC1. This highlights the substantive movement of the dimeric 2x(HhH)2 domain and the rotation by 15[illegible] of the XPF helical domain away from the XPF nuclease domain. The main conformational changes occur at the autoinhibition interfaces.
