# Supplementary material for: Cryo-EM structures of the XPF-ERCC1 endonuclease reveal how DNA-junction engagement disrupts an auto-inhibited conformation
Source: Nat Commun. 2020 Feb 28;11:1120. doi: 10.1038/s41467-020-14856-2 (PMC7048804; doi:10.1038/s41467-020-14856-2)
Supplement: Supplementary file 13 — Reporting Summary [file 41467_2020_14856_MOESM13_ESM.pdf]

nature research

Corresponding author(s): Neil Q McDonald

Last updated by author(s): Jan 30, 2020

Reporting Summary

Nature Research wishes to improve the reproducibility of the work that we publish. This form provides structure for consistency and transparency in reporting. For further information on Nature Research policies, see Authors & Referees and the Editorial Policy Checklist .

Statistics

For all statistical analyses, confirm that the following items are present in the figure legend, table legend, main text, or Methods section.

n/a	Confirmed	
☐	☒	The exact sample size (*n*) for each experimental group/condition, given as a discrete number and unit of measurement
☐	☒	A statement on whether measurements were taken from distinct samples or whether the same sample was measured repeatedly
☒	☐	The statistical test(s) used AND whether they are one- or two-sided<br>*Only common tests should be described solely by name; describe more complex techniques in the Methods section.*
☒	☐	A description of all covariates tested
☒	☐	A description of any assumptions or corrections, such as tests of normality and adjustment for multiple comparisons
☐	☒	A full description of the statistical parameters including central tendency (e.g. means) or other basic estimates (e.g. regression coefficient) AND variation (e.g. standard deviation) or associated estimates of uncertainty (e.g. confidence intervals)
☒	☐	For null hypothesis testing, the test statistic (e.g. *F*, *t*, *r*) with confidence intervals, effect sizes, degrees of freedom and *P* value noted<br>*Give P values as exact values whenever suitable.*
☒	☐	For Bayesian analysis, information on the choice of priors and Markov chain Monte Carlo settings
☒	☐	For hierarchical and complex designs, identification of the appropriate level for tests and full reporting of outcomes
☒	☐	Estimates of effect sizes (e.g. Cohen's *d*, Pearson's *r*), indicating how they were calculated

Our web collection on statistics for biologists contains articles on many of the points above.

Software and code

Policy information about availability of computer code

Data collection	EPU (Thermo-Fisher), Reader control software (BMG Labtech),
Data analysis	MARS data analysis (BMG Labtech), Xmipp-3.0, Relion-2.0, Relion-3.0, EMAN2, Gautomatch, ctffind4, MotionCor2, UCSF Chimera (RBVI), Coot-0.8.9, Flex-EM, Phenix-1.16, cryoSPARC-1 (Structura Biotechnology Inc.), cryoSPARC-2 (Structura Biotechnology Inc.), Phyre2, MolProbity, Scipion-1.2, BlocRes, Proteome Discoverer-2.2 (Thermo-Fisher), PRISM-7 (GraphPad)

For manuscripts utilizing custom algorithms or software that are central to the research but not yet described in published literature, software must be made available to editors/reviewers. We strongly encourage code deposition in a community repository (e.g. GitHub). See the Nature Research guidelines for submitting code & software for further information.

Data

Policy information about availability of data

All manuscripts must include a data availability statement. This statement should provide the following information, where applicable:

- Accession codes, unique identifiers, or web links for publicly available datasets
- A list of figures that have associated raw data
- A description of any restrictions on data availability

The XPF-ERCC1 structure has been deposited in the Protein Data Bank (www.pdb.org) with the primary accession code 6SXA and EMDB code EMD-10337. The XPF-ERCC1-dsDNA structure has been deposited in the Protein Data Bank (www.pdb.org) with the primary accession code 6SXB and EMDB entry code EMD-10338. All data supporting the findings of this study are available from the corresponding author upon request.

Field-specific reporting

Please select the one below that is the best fit for your research. If you are not sure, read the appropriate sections before making your selection.

[x] Life sciences   [ ] Behavioural & social sciences   [ ] Ecological, evolutionary & environmental sciences

For a reference copy of the document with all sections, see nature.com/documents/nr-reporting-summary-flat.pdf

Life sciences study design

All studies must disclose on these points even when the disclosure is negative.

Sample size	The number of independent experiments (n) performed for each assay is indicated in the figures. Each experiment was performed three times. No sample size calculation was done for this study. Standard deviation was calculated for each replicate group using GraphPad PRISM-7. No statistical tests were carried out for this study.
Data exclusions	Recombinant mutants of XPF and ERCC1 were only assayed if it was possible to recover a sufficient amount of pure protein that was detectable on a size-exclusion chromatography trace.
Replication	In-vitro activity assays were successfully repeated independently three times, as indicated in the figures (n experiments).
Randomization	Particle images were randomly assigned to half sets in order to determine the gold standard FSC resolution.
Blinding	The data collection and analyses were done blindly when the experiments allowed.

Reporting for specific materials, systems and methods

We require information from authors about some types of materials, experimental systems and methods used in many studies. Here, indicate whether each material, system or method listed is relevant to your study. If you are not sure if a list item applies to your research, read the appropriate section before selecting a response.

Materials & experimental systems

n/a	Involved in the study	
[ ]	[x]	Antibodies
[x]	[ ]	Eukaryotic cell lines
[x]	[ ]	Palaeontology
[x]	[ ]	Animals and other organisms
[x]	[ ]	Human research participants
[x]	[ ]	Clinical data

Methods

n/a	Involved in the study	
[x]	[ ]	ChIP-seq
[x]	[ ]	Flow cytometry
[x]	[ ]	MRI-based neuroimaging

Antibodies

Antibodies used	Anti-SLX4 (generated in-house), goat anti mouse, HRP (Thermo-Fisher. Catalog # 31430)
Validation	Anti-SLX4 antibody was generated in-house by the West lab. Used in: Wyatt HD, Laister RC, Martin SR, Arrowsmith CH, West SC. The SMX DNA Repair Tri-nuclease. Mol Cell. 2017;65(5):848–860.e11. doi:10.1016/j.molcel.2017.01.031.
