# Supplementary material for: Cryo-EM structures of the XPF-ERCC1 endonuclease reveal how DNA-junction engagement disrupts an auto-inhibited conformation
Source: Nat Commun. 2020 Feb 28;11:1120. doi: 10.1038/s41467-020-14856-2 (PMC7048804; doi:10.1038/s41467-020-14856-2)

Figure 1b

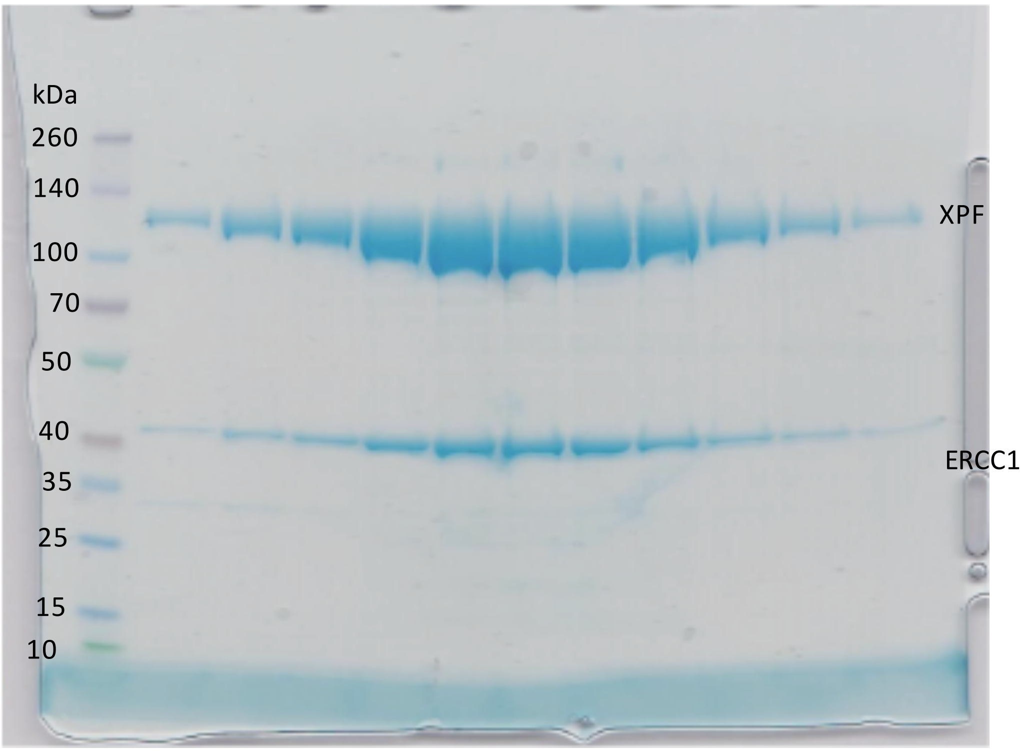

Figure 5b

WT XPF-ERCC1	Vmax (fmol/min) stem loop DNA	ssDNA	dsDNA	ss + dsDNA
Replicate #1	99.3	1.32	0.13	2.01
Replicate #2	106.4	-0.323	0.03	0.04
Replicate #3	112.1	2.22	-0.145	4.12

Supplementary Figure 1b

WT XPF-ERCC1	Vmax (fmol/min) stem loop DNA
Replicate #1	88.6
Replicate #2	110.5
Replicate #3	102.4

Supplementary Figure 9d

XPF-ERCC1-SLX4(NTD)	Vmax (fmol/min) stem loop DNA
Replicate #1	228.7
Replicate #2	217.6
Replicate #3	209.7

Supplementary Figure 9e

Anti - SLX4 antibody

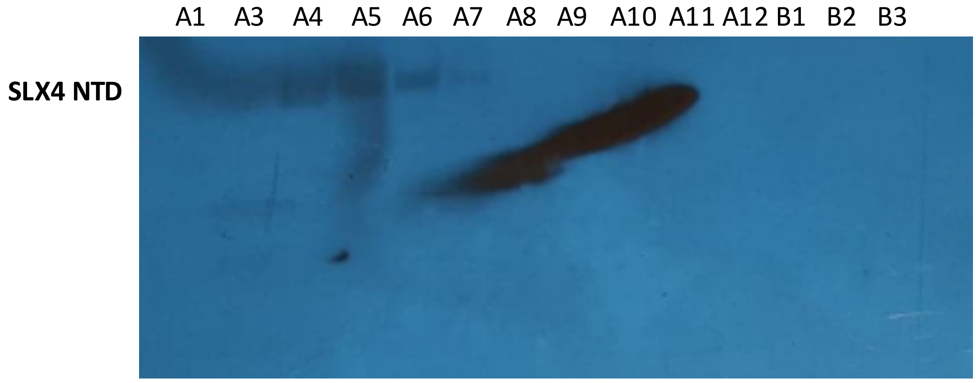

Anti - XPF antibody

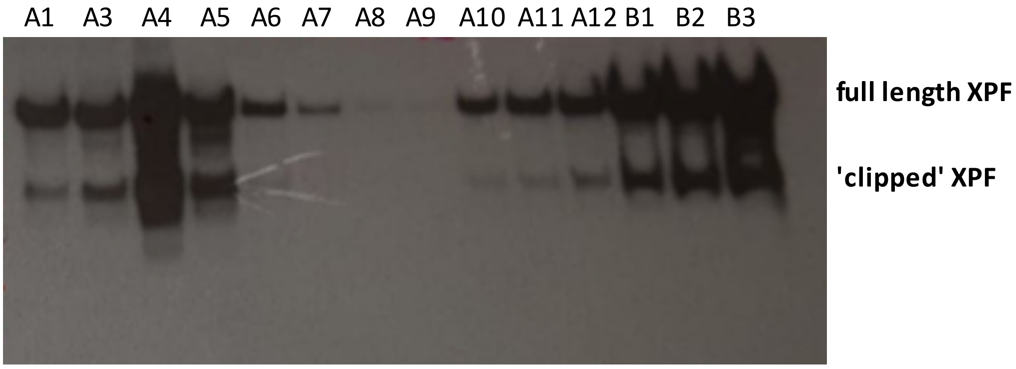

Supplement: Supplementary file 14 — Source Data [file 41467_2020_14856_MOESM14_ESM.pdf]
